# Adenosine A2B receptor activation regulates the balance between T helper 17 cells and regulatory T cells, and inhibits regulatory T cells exhaustion in experimental autoimmune myositis

**DOI:** 10.1002/jcsm.13581

**Published:** 2024-09-16

**Authors:** Yueyuan Zhou, Limei Kang, Geng Yin, Leiyi Yang, Bo Chen, Binhan Liu, Xiaoyan Zhu, Qibing Xie

**Affiliations:** ^1^ Department of Rheumatology and Immunology West China Hospital, Sichuan University Chengdu Wuhou District China; ^2^ Department of General Practice, General Practice Medical Center West China Hospital, Sichuan University Chengdu Wuhou District China; ^3^ Department of Physiology Naval Medical University Shanghai Yangpu District China

**Keywords:** Adenosine A2B receptor, Experimental autoimmune myositis, Hypoxia inducible factor‐1α, Idiopathic inflammatory myopathy, Tregs exhaustion

## Abstract

**Background:**

Idiopathic inflammatory myopathy (IIM) is a systemic autoimmune disease characterized by skeletal muscle involvement. This study aimed to investigate the role of adenosine receptor signalling pathways in the development of experimental autoimmune myositis (EAM).

**Methods:**

An ecto‐5′‐nucleotidase (CD73) inhibitor, adenosine receptor agonists, a hypoxia‐inducible factor‐1α (HIF‐1α) inhibitor or a vehicle were administered to control and EAM mice. Murine splenic CD4^+^ or regulatory T cells (Tregs) were isolated using magnetic beads and subsequently stimulated with an adenosine A2B receptor agonist, a HIF‐1α inhibitor, or vehicle in vitro. In cross‐sectional studies, we collected 64 serum samples (69% female, 49 ± 9 years), 63 peripheral blood samples (70% female, 50 ± 11 years), and 34 skeletal muscle samples (71% female, 63 ± 6 years) from patients with IIM. Additionally, 35 serum samples and 30 peripheral blood samples were obtained from age‐ and sex‐matched healthy controls, and six quadriceps muscle samples were collected from patients with osteoarthritis to serve as the normal group.

**Results:**

Patients with IIM exhibited increased CD73 [dermatomyositis (DM), polymyositis (PM): *P* < 0.01; immune‐mediated necrotizing myopathy (IMNM): *P* < 0.0001] and adenosine deaminase (ADA) expression (DM: *P* < 0.001; PM, IMNM: *P* < 0.0001) in the skeletal muscles, and serum ADA levels [56.7 (95% CI: 53.7, 58.7) vs. 198.8 (95% CI: 186.2, 237.3) ng/μL, *P* < 0.0001]. Intervention with a CD73 inhibitor exacerbated (*P* = 0.0461), whereas adenosine receptor agonists (A1: *P* = 0.0009; A2B: *P* < 0.0001; A3: *P* = 0.0001) and the HIF‐1α inhibitor (*P* = 0.0044) alleviated skeletal muscle injury in EAM mice. Elevated expression of programmed cell death protein‐1 (PD1: *P* = 0.0023) and T‐cell immunoglobulin and mucin‐domain containing‐3 (TIM3: *P* < 0.0001) in skeletal muscles of patients with IIM were correlated with creatine kinase levels (PD1, *r* = 0.7072, *P* < 0.0001; TIM3, *r* = 0.4808, *P* = 0.0046). PD1^+^CD4^+^ (*r* = 0.3243, *P* = 0.0115) and PD1^+^CD8^+^ (*r* = 0.3959, *P* = 0.0017) T cells were correlated with Myositis Disease Activity Assessment Visual Analogue Scale scores (muscle) in IIM. The exhausted Tregs were identified in the skeletal muscles of patients with IIM. Activation of the A2B adenosine receptor downregulated HIF‐1α (protein or mRNA level, *P* < 0.01), resulting in decreased T helper cell 17 (Th17) (13.58% vs. 5.43%, *P* = 0.0201) and phosphorylated‐signal transducer and activator of transcription 3 (p‐STAT3)^+^ Th17 (16.32% vs. 6.73%, *P* = 0.0029), decreased exhausted Tregs (PD1^+^ Tregs: 53.55% vs. 40.28%, *P* = 0.0005; TIM3^+^ Tregs: 3.93% vs. 3.11%, *P* = 0.0029), and increased Tregs (0.45% vs. 2.89%, *P* = 0.0006) in EAM mice.

**Conclusions:**

The exhausted T cells may be pathogenic in IIM, and the activation of adenosine A2B receptor signalling pathway can regulate Th17/Treg balance and inhibit Tregs exhaustion, thereby slowing EAM disease progression.

## Introduction

Idiopathic inflammatory myopathy (IIM) is a group of systemic autoimmune diseases characterized by skeletal muscle involvement. The main subtypes of IIM include dermatomyositis (DM), polymyositis (PM), immune‐mediated necrotizing myopathy (IMNM), inclusion body myositis, and antisynthetase syndrome. IIM predominantly affects middle‐aged and older populations, with a slightly higher prevalence in females than that in males.^1^ This condition can significantly reduce patients' quality of life, especially when life‐threatening complications involve vital organs.^2^ However, the precise pathogenesis underlying IIM remains unclear. Therefore, and investigating pathogenesis of IIM and exploring new therapeutic targets is essential.

Various immunosuppressants effectively treat rheumatic immune diseases through elevating adenosine levels.^3^ However, the role of adenosine and its signalling pathways in IIM remains unknown. Adenosine is a widely present nucleoside in living organisms. Extracellular enzymes, including ectonucleoside triphosphate diphosphohydrolase‐1 (NTPDase‐1, CD39) and ecto‐5′‐nucleotidase (NT5E, which belong to CD73), catalyse adenosine generation from AMP and ADP. Extracellular adenosine deaminase (ADA) converts adenosine into inosine. Adenosine receptors, the G protein‐coupled receptor class, are distributed throughout the human body and can be categorized into four subtypes: A1, A2A, A2B, and A3. Adenosine binds to membrane‐bound receptors and participates in diverse physiological and pathological processes, including cellular metabolism, tissue repair, and inflammatory and immune regulation.^4^

Adenosine receptor signalling pathways are widely known to be involved in lymphocyte differentiation, activity, and functional regulation.^5^ Chronic muscle inflammation is a hallmark of IIM.^6^ T cell‐mediated inflammation and immune dysfunction, particularly Type 1 helper T cells (Th1) and Th17 secrete interferon‐gamma (IFN‐γ) and interleukin (IL)‐17, involved in the inflammatory response in the affected tissue.^7^, ^8^ Decreased regulatory T cells (Tregs) and their dysfunction contribute to immune imbalance and autoimmune responses in IIM.^9^ Therefore, it is worth exploring whether activation of adenosine receptor signalling pathways is involved in the development of EAM by regulating T cell differentiation and function.

Studies have shown that the adenosine receptor signalling pathways, including CD39 and CD73 (as new immune checkpoints), are correlated with T cell exhaustion, but these studies were mainly focused on tumours.^10^, ^11^ However, whether the adenosine receptor signalling pathway contributes to the development of autoimmune diseases by affecting T cell exhaustion remains unknown. Prolonged exposure to antigens and inflammatory environments leads to continuous stimulation of T cells, resulting in a gradual decline in effector function and memory T cell characteristics, called T cell exhaustion. T cell exhaustion is characterized by effector function loss, persistent increased expression of inhibitory receptors, epigenetic and transcriptional profile changes, and altered metabolism.^12^, ^13^ Currently, the characteristics of T cell exhaustion and its role in IIM remain unclear. Continuous antigen exposure may induce T cell exhaustion in inclusion body myositis and IMNM.^14^ Numerous case reports of immune checkpoint inhibitor‐related myositis (immune‐related adverse events) have recently emerged, suggesting that inhibition of T‐cell exhaustion may activate autoimmune reactions.^15^ Conversely, another study demonstrated that programmed cell death 1 (PD‐1) positive CD8^+^ cells with high expression of cytolytic molecules are the pathogenic cell subset in IIM.^16^ Therefore, investigating the features and role of exhausted T cells in IIM and evaluating the potential impact of the adenosine receptors signalling pathway on T cell exhaustion is necessary.

The experimental autoimmune myositis (EAM) is a well‐established animal model used to study the immunopathogenesis of IIM.^17^ Here, we investigated the roles of the adenosine receptor signalling pathways in the progression of EAM, which will provide novel insights into the pathogenesis of IIM and theoretical support for new treatment strategies.

## Materials and methods

### Clinical sample collection

In our cross‐sectional studies, we collected 64 serum samples, 63 peripheral blood mononuclear cell samples, and 34 skeletal muscle specimens from patients with IIM at the West China Hospital, Sichuan University (6/2021–10/2023). Peripheral blood samples were collected from 35 (for serological detection) or 30 (for flow cytometry detection) age‐ and sex‐matched healthy controls (HCs). Additionally, quadriceps muscle specimens obtained during knee joint replacement surgery from 6 patients with osteoarthritis served as the normal group (NG). The diagnostic criteria, exclusion criteria, and clinical information are shown in Data S2 and Data S1 (Tables S1–S3). This study was approved by the Ethics Committee of the West China Hospital, Sichuan University (No. 521, 2021), Written informed consent was obtained from all participants. and complied with the principles of the Declaration of Helsinki.

### Procedures for animal administration

Eight‐week‐old female BALB/c mice (weighing approximately 18–22 g) were purchased from the HFKbio company (Beijing, China) and housed in the animal care facility of the West China Hospital. All protocols involving animals were reviewed and approved by the institutional animal welfare committee of West China Hospital, Sichuan University (No. 20220808002). Construction methods for the EAM model are detailed in Supplementary Material 2.2.2.

On the 8th day of modelling, mice received daily intraperitoneal injections of 100 μL saline or CD73 inhibitor (5 mg/kg/day)^18^ for ten consecutive days. Additionally, on the 8th day of modelling, the control and EAM model groups received daily intraperitoneal injections of 100 μL of either vehicle (5% DMSO + 40% Polyethylene glycol 300 + 5% Polysorbate 80 + 50% ddH2O) or N6‐‐Cyclopentyladenosine (adenosine A1 receptor agonist, 100 μg/kg/day),^19^ CGS21680 HCl (adenosine A2A receptor agonist, 0.5 mg/kg/day),^20^ BAY60‐6583 (adenosine A2B receptor agonist, 2 mg/kg/day),^21^ or Namodenoson (adenosine A3 receptor agonist, 100 μg/kg/day),^22^ respectively, for 14 consecutive days. Furthermore, on the 8th day of modelling, mice received daily intraperitoneal injections of 200 μL of either vehicle or HIF‐1α inhibitor (LW6, 2 mg/kg/day)^23^ for 14 consecutive days. For details, see Data S2.

### Cell stimulation

Sorted CD4^+^ T cells or Tregs were cultured in a 12‐well plate and received DMSO, or A2B adenosine receptor agonist (5 μM),^24^ or HIF‐1α inhibitor (10 μM).^25^ Cells and culture supernatants were collected after 12 hours of stimulation for subsequent experiments.

### Others

The skeletal muscle injury scoring criteria, GSE datasets of IIM, and primer sequences are listed in Data S1 (Tables S4–S6). Details of the assessment of muscle strength, immunohistochemical staining, enzyme‐linked immunosorbent assay (ELISA) detection, western blot, quantitative polymerase chain reaction, T lymphocyte sorting, flow cytometry staining, eukaryotic mRNA sequencing, bioinformatics analysis, statistical analysis, and other methods are provided in Data S2.

## Results

### CD73 inhibition accelerates the progression of EAM, and serum ADA levels are correlated with muscle enzymes in IIM

**Figure 1:**
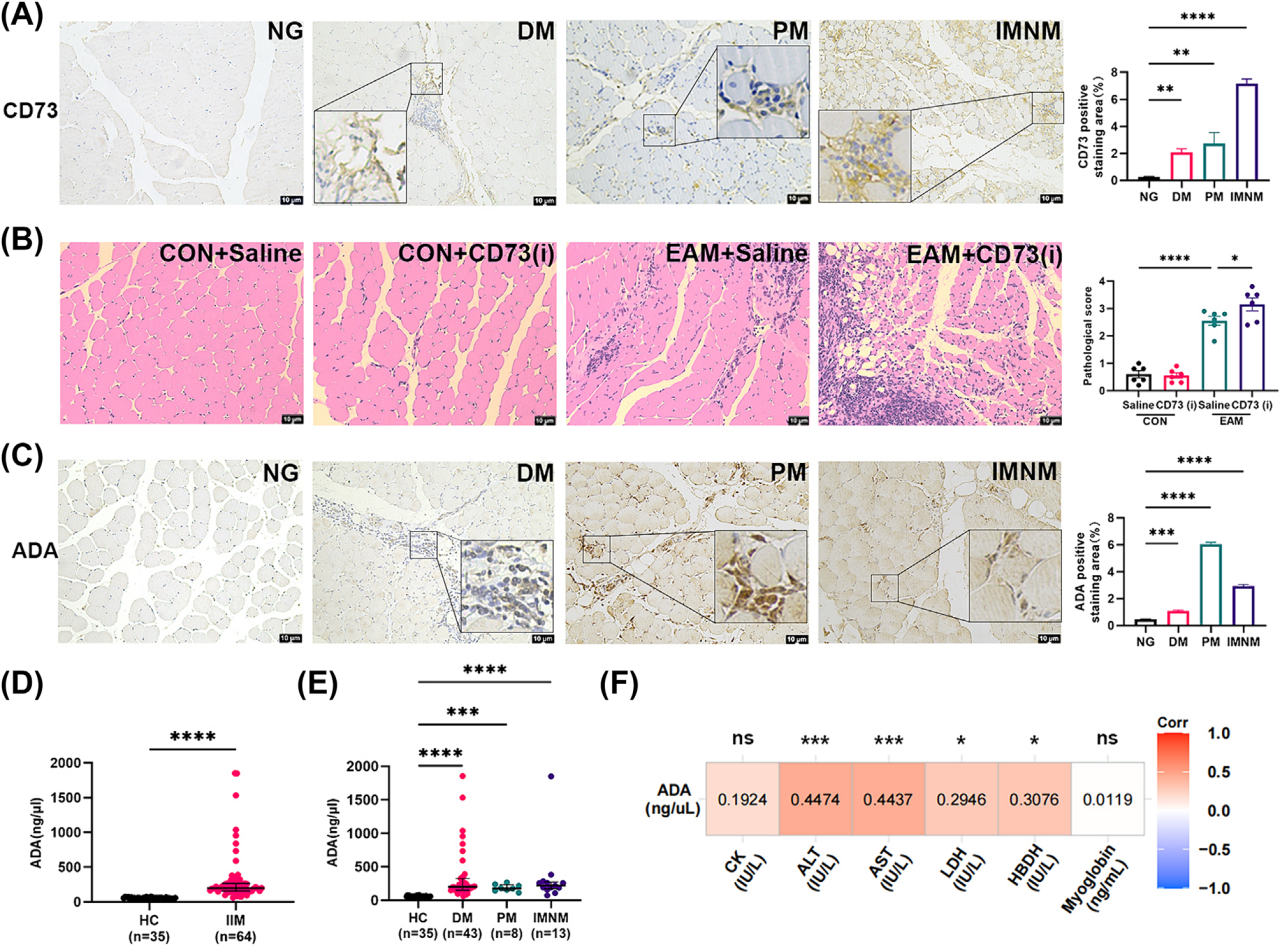
CD73 inhibition accelerates the progression of EAM, and serum ADA levels correlate with muscle enzymes in IIM. (A) Immunohistochemical staining of CD73 in skeletal muscle of patients with IIM (10 DM, 3 PM, 10 IMNM) and its semiquantitative analysis vs. NG (*n* = 6) group. (B) Hematoxylin and Eosin staining and pathological damage and inflammation scores of mouse skeletal muscle, *n* = 5–6; vs. EAM + Saline group. (C) Immunohistochemical staining of ADA in skeletal muscle of patients with IIM (10 DM, 3 PM, 10 IMNM) and its semi‐quantitative analysis. vs. NG (*n* = 6) group. Serum ADA levels in HC and IIM (D) or subtypes of IIM (E). vs. HC group. (F) The heat map of correlation analysis between serum ADA levels and ALT, AST, CK, LDH, HBDH, and myoglobin levels in patients with IIM (*n* = 64). The numbers in the coloured squares are the values of the correlation coefficient (*r)*. ADA: adenosine deaminase; ALT: alanine aminotransferase; AST: aspartate aminotransferase; CK: creatine kinase; DM: dermatomyositis; HBDH: hydroxybutyrate dehydrogenase; HC: healthy control; IMNM: immune‐mediated necrotizing myopathy; JDM: juvenile dermatomyositis; LDH: lactate dehydrogenase; NG: normal group; PM: polymyositis. Scale bar = 10 μm; *P < 0.05, ** *P* < 0.01, ***P < 0.001, ****P < 0.0001.

The mRNA levels of CD73 were higher in the skeletal muscles of patients with DM (GSE128470, GSE48280, GSE1551: *P* < 0.01; GSE5370: *P* < 0.05) than in those of the NG. Similar elevations were observed in juvenile dermatomyositis (JDM) (GSE11971, *P* < 0.01; GSE3307, *P* < 0.0001) and PM (GSE128470, *P* < 0.05) (Figure S1A). CD39 expression showed no significant differences in the skeletal muscles of patients with IIM compared with NG (Figure S1B). Immunohistochemical staining confirmed higher CD73 expression in the skeletal muscles of patients with DM (*P* < 0.01), PM (*P* < 0.01), and IMNM (*P* < 0.0001), predominantly in infiltrating inflammatory cells (Figure 1A).

EAM mice exhibited higher inflammatory cell infiltration and greater muscle fibre damage (*P* < 0.0001, Figure 1B), significant weight loss (*P* = 0.0004), spleen weight increase (*P* < 0.0001), and decreased limb muscle strength (118.40 vs. 102.60 g, *P* = 0.0093) compared with controls (Figure S2). Following CD73 inhibitor administration, muscle damage and inflammatory infiltration increased (*P* = 0.0461, Figure 1B), spleen weight increased (*P* = 0.0184), and muscle strength declined (102.60 vs. 84.73 g, *P* = 0.0036) (Figure S2A–C) in EAM mice. Serum IL‐1β (4.33 vs. 17.66 pg/mL, *P* < 0.0001), TNF‐α (44.40 vs. 115.70 pg/mL, *P* < 0.0001), IL‐6 (11.42 vs. 35.78 pg/mL, *P* < 0.0001), IL‐2 (61.20 vs. 82.66 pg/mL, *P* = 0.0164), and IFN‐γ (147.40 vs. 205.10 pg/mL, *P* = 0.0228) levels increased in EAM compared with controls, while EAM mice exhibited increased serum IL‐1β (17.66 vs. 23.76 pg/mL, *P* = 0.0214) and IFN‐γ (205.10 vs. 263.90 pg/mL, *P* = 0.0273) levels after CD73 inhibitor intervention. However, serum TNF‐α, IL‐6, and IL‐2 levels showed no significant change (Figure S2D–H).

Patients with DM (*P* < 0.001), PM (*P* < 0.0001), and IMNM (*P* < 0.0001) showed higher ADA expression in skeletal muscles than NG (Figure 1C). ELISA results revealed a significant increase in serum ADA levels in patients with IIM compared with HCs [56.7 (95% CI: 53.7, 58.7) vs. 198.8 (95% CI: 186.2, 237.3) ng/μL, *P* < 0.0001, Figure 1D]. The greatest increases were observed in patients with DM [56.7 vs. 199.7 (95% CI: 185.3, 242.7) ng/μL, *P* < 0.0001], PM [56.7 vs. 176.3 (95% CI: 110.9, 265.1) ng/μL, *P* = 0.0006], and IMNM [56.7 vs. 217.6 (95% CI: 158.4, 283.1) ng/μL, *P* < 0.0001] (Figure 1E). Furthermore, our findings indicated a positive correlation between serum ADA levels and ALT (*r* = 0.4474; *P* = 0.0002), AST (*r* = 0.4437; *P* = 0.0002), LDH (*r* = 0.2946; *P* = 0.0181), and HBDH (*r* = 0.3076; *P* = 0.0134), with no significant correlation observed with CK (*r* = 0.1924; *P* = 0.1277) and myoglobin (*r* = 0.0119; *P* = 0.9276) levels (Figure 1F).

### Adenosine receptor agonists attenuate skeletal muscle injury and inflammation

**Figure 2:**
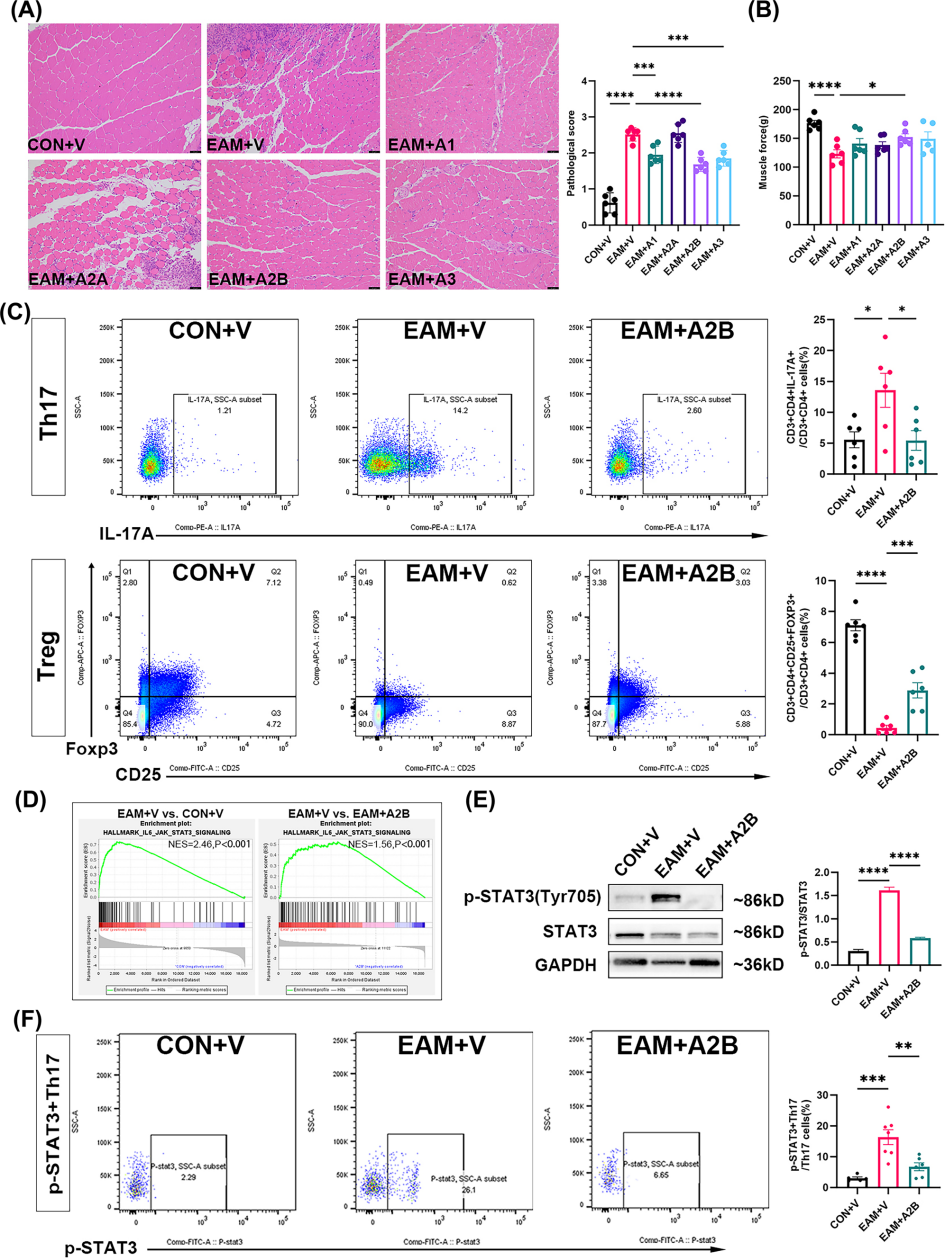
Activation of adenosine A2B receptor slows down the progression of EAM by promoting Tregs differentiation and inhibiting Th17 differentiation. (A) Hematoxylin and Eosin staining and pathological damage and inflammation scores of mouse skeletal muscle. (B) Muscle force of mice. (C) Flow scatter plots and proportion of splenic Th17 and Treg. (D) GSEA enrichment analysis of IL‐6/STAT3 signalling pathway in mouse skeletal muscle. (E) Representative immunoblot bands and grey value analysis of p‐STAT3 and STAT3 in mouse skeletal muscle. (F). Flow scatter plots and proportion of splenic p‐STAT3^+^Th17. Scale bar = 50 μm, *n* = 5–7. CON: control; EAM: experimental autoimmune myositis; V: vehicle; GSEA: gene set enrichment analysis; vs. EAM + V group, *P < 0.05, **P < 0.01, ***P < 0.001, ****P < 0.0001.

Adenosine receptor agonists (A1, A2A, A2B, A3) were used to intervene in EAM mice. As shown in Figure 2A, the pathological injury score of the skeletal muscle was higher in the EAM model group than that in the controls (*P* < 0.0001). However, following adenosine A1, A2B, and A3 receptor agonist administration, the infiltrated inflammation and injury in the affected skeletal muscles of EAM mice significantly decreased, leading to a significant reduction in the pathological injury score (A1, *P* = 0.0009; A2B, *P* < 0.0001; A3, *P* = 0.0001). The EAM mice showed reduced body weight (20.33 vs. 18.30 g, *P* = 0.0017, Figure S3A), increased spleen weight (0.10 vs. 0.22 g, *P* < 0.0001, Figure S3B), and a significant decrease in limb muscle strength compared with the controls (176.50 vs. 123.00 g, *P* < 0.0001, Figure 2B). Muscle strength partly recovered only after A2B agonist administration (123.00 vs. 152.00 g, *P* = 0.0382, Figure 2B). As shown in Figure S3C–G, EAM mice exhibited elevated levels of serum IL‐1β (6.93 vs. 23.14 pg/mL, *P* < 0.0001), TNF‐α (40.51 vs. 90.98 pg/mL, *P* = 0.0014), IL‐6 (25.85 vs. 35.04 pg/mL, *P* = 0.0207), IL‐2 (52.35 vs. 79.13 pg/mL, *P* = 0.0028), and IFN‐γ (135.40 vs. 273.80 pg/mL, *P* = 0.0016), compared with the controls. Following intervention with adenosine A1, A2B, and A3 receptor agonists, serum IL‐1β (23.14 vs. 15.15 pg/mL, *P* = 0.0039; vs. 17.02 pg/mL, *P* = 0.0244, vs. 15.20 pg/mL, *P* = 0.0026), IL‐6 (35.04 vs. 25.27 pg/mL, *P* = 0.0129; vs. 23.02 pg/mL, *P* = 0.0012; vs. 24.60 pg/mL, *P* = 0.0049), and IL‐2 (79.13 vs. 51.60, *P* = 0.0022; vs. 58.53, *P* = 0.0184; vs. 59.58, *P* = 0.0267) levels decreased. Additionally, only intervention with the adenosine A2B receptor agonist reduced serum TNF‐α (90.98 vs. 59.68 pg/mL, *P* = 0.0477) and IFN‐γ (273.80 vs. 180.20 pg/mL, *P* = 0.0303) levels in EAM mice.

As shown in Figure 2C, compared with the controls, the splenic Th17 cell proportion significantly increased in EAM mice (5.55% vs. 13.58%, *P* = 0.0219) and decreased after adenosine A2B receptor agonist administration (13.58% vs. 5.43%, *P* = 0.0201). The splenic Tregs proportion decreased significantly in EAM mice (7.11% vs. 0.45%, *P* < 0.0001) and increased after adenosine A2B receptor agonist administration (0.45% vs. 2.89%, *P* = 0.0006). The splenic CD8^+^ T cell proportion significantly decreased in EAM mice (6.37% vs. 2.80%, *P* = 0.0033); however, there were no significant changes in Th1, Th2, and CD8^+^ T cells after adenosine A2B receptor agonist administration (Figure S4).

### The adenosine A2B receptor agonist downregulates Th17 cell proportion by suppressing signal transducer and activator of transcription 3 (STAT3) phosphorylation

Gene set enrichment analysis (GSEA) revealed upregulated IL‐6/STAT3 signalling in IIM (PM, DM, and IMNM, Figure S5A–D) and EAM (*P* < 0.001, Figure 2D), and this pathway was downregulated after adenosine A2B receptor agonist intervention (*P* < 0.001) (Figure 2D).

Additionally, the p‐STAT3/STAT3 protein ratio in skeletal muscles of EAM mice significantly exceeded that of the controls (*P* < 0.0001) and significantly decreased after the adenosine A2B receptor agonist intervention (*P* < 0.0001) (Figure 2E). Furthermore, we observed a significant increase in splenic phosphorylated‐STAT3 (p‐STAT3)^+^ Th17 in EAM mice compared with that in the controls (3.00% vs. 16.32%, *P* = 0.0003), which was reduced after intervention with the adenosine A2B receptor agonist (16.32% vs. 6.73%, *P* = 0.0029) (Figure 2F).

### The adenosine A2B receptor agonist inhibits CD4^+^ T‐cell exhaustion

**Figure 3:**
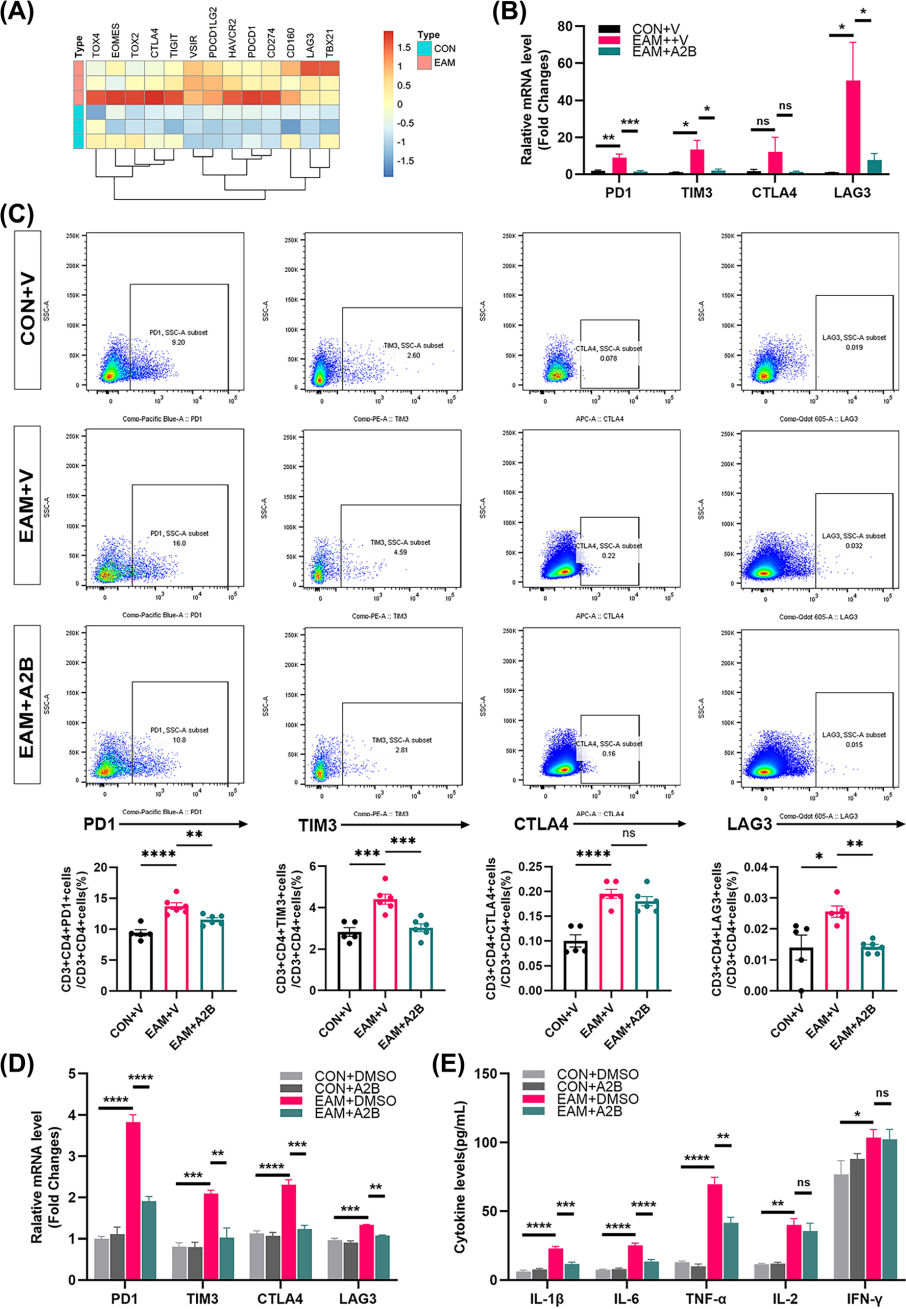
Activation of adenosine A2B receptors inhibits CD4^+^T cell exhaustion in vivo and in vitro. (A) Heat map of exhaustion‐related gene expression in mouse skeletal muscle transcriptome sequencing (CON + V vs. EAM + V; *n* = 3). (B) mRNA levels of PD1, TIM3, CTLA4, and LAG3 in mouse skeletal muscle; *n* = 5–6. (C) Flow scatter plots and proportions of exhausted (PD1^+^CD4^+^ T cells, TIM3^+^CD4^+^ T cells, CTLA4^+^CD4^+^ T cells, and LAG3^+^CD4^+^ T cells) T cells. (D) mRNA levels of PD1, TIM3, CTLA4, and LAG3 in spleen CD4^+^ T cells stimulated with DMSO or adenosine A2B receptor agonist. (E) Cell supernatant levels of interleukin (IL)‐1β, IL‐6, TNF‐α, IL‐2, and IFN‐γ; *n* = 3. CON: control; CTLA4: cytotoxic T‐lymphocyte‐associated protein‐4; EAM: experimental autoimmune myositis; IFN‐γ: interferon‐gamma; LAG3: lymphocyte‐activation gene‐3; PD1: programmed cell death protein‐1; TIM3: T‐cell immunoglobulin and mucin‐domain containing‐3; TNF‐α: tumour necrosis factor‐gamma; V: vehicle. vs. EAM + V group or EAM + DMSO group, *P < 0.05, **P < 0.01, ***P < 0.001, ****P < 0.0001.

As shown in the heat map (Figure 3A), an increasing trend in the expression of T‐cell exhaustion‐related genes was observed in the skeletal muscles of EAM mice compared with controls. The skeletal muscle of EAM mice exhibited higher mRNA levels of PD1 (*P* = 0.0013), TIM3 (*P* = 0.0101), and LAG3 (*P* = 0.0185) than those of the controls, which were decreased following treatment with the adenosine A2B receptor agonist (PD1, *P* = 0.0008; TIM3, *P* = 0.0161; LAG3, *P* = 0.0321; Figure 3B). An increased proportion of splenic CD4^+^PD1^+^ T cells (9.40% vs. 13.72%, *P* < 0.0001), CD4^+^TIM3^+^ T cells (2.82% vs. 4.41%, *P* = 0.0003), CD4^+^LAG3^+^ T cells (0.0140% vs. 0.0256%, *P* = 0.0110), and CD4^+^CTLA4^+^ T cells (0.0998% vs. 0.1950%, *P* < 0.0001) were observed compared with the controls. After intervention with an adenosine A2B receptor agonist, the proportion of splenic CD4^+^PD1^+^ T cells (13.72% vs. 11.52%, *P* = 0.0085), CD4^+^TIM3^+^ T cells (4.41% vs. 3.03%, *P* = 0.0007), and CD4^+^LAG3^+^ T cells (0.0256% vs. 0.0142%, *P* = 0.0092) decreased (Figure 3C). Additionally, the proportion of splenic CD8^+^PD1^+^ T cells (1.86% vs. 3.07%, *P* = 0.0131), CD8^+^TIM3^+^ T cells (1.39% vs. 1.90%, *P* = 0.0179), and CD8^+^LAG3^+^ T cells (0.06% vs. 0.23%, *P* = 0.0023) increased compared with the controls. Only the proportion of CD8^+^PD1^+^ T cells decreased after intervention with the adenosine A2B receptor agonist (3.07% vs. 2.10%, *P* = 0.0353) (Figure S6).

CD4^+^ T cells sorted from the spleens of control and EAM mice were stimulated in vitro with the adenosine A2B receptor agonist or DMSO. As shown in Figure 3D, compared with the control (CON+DMSO) cells, the mRNA levels of PD1 (*P* < 0.0001), TIM3 (*P* = 0.0007), CTLA4 (*P* < 0.0001), and LAG3 (*P* = 0.0001) were significantly increased in the CD4^+^ T cells of the EAM mice (EAM + DMSO). However, after stimulation with the adenosine A2B receptor agonist, the mRNA expression levels of PD1 (*P* < 0.0001), TIM3 (*P* = 0.0022), CTLA4 (*P* = 0.0001), and LAG3 (*P* = 0.0015) in the CD4^+^ T cells of the EAM mice significantly decreased. As shown in Figure 3E, compared with the controls cells, the culture supernatant of model CD4^+^ T cells exhibited increased levels of IL‐1β (6.31 vs. 22.90 pg/mL, *P* < 0.0001), IL‐6 (7.43 vs. 25.21 pg/mL, *P* < 0.0001), TNF‐α (13.11vs. 69.58 pg/mL, *P* < 0.0001), IL‐2 (11.48 vs. 40.03 pg/mL, *P* = 0.0014), and IFN‐γ (76.89 vs. 103.50 pg/mL, *P* = 0.0319). After stimulation with the adenosine A2B receptor agonist, the culture supernatant of model CD4^+^ T cells showed a decrease in IL‐1β (22.90 vs. 11.63 pg/mL, *P* = 0.0001), IL‐6 (25.21 vs. 13.62 pg/mL, *P* < 0.0001), and TNF‐α (69.5 vs. 41.38 pg/mL, *P* = 0.0019) levels, whereas the IL‐2 and IFN‐γ levels showed no significant differences.

### Exhausted CD4^+^ and CD8^+^ T cells are positively correlated with disease activity in IIM

**Figure 4:**
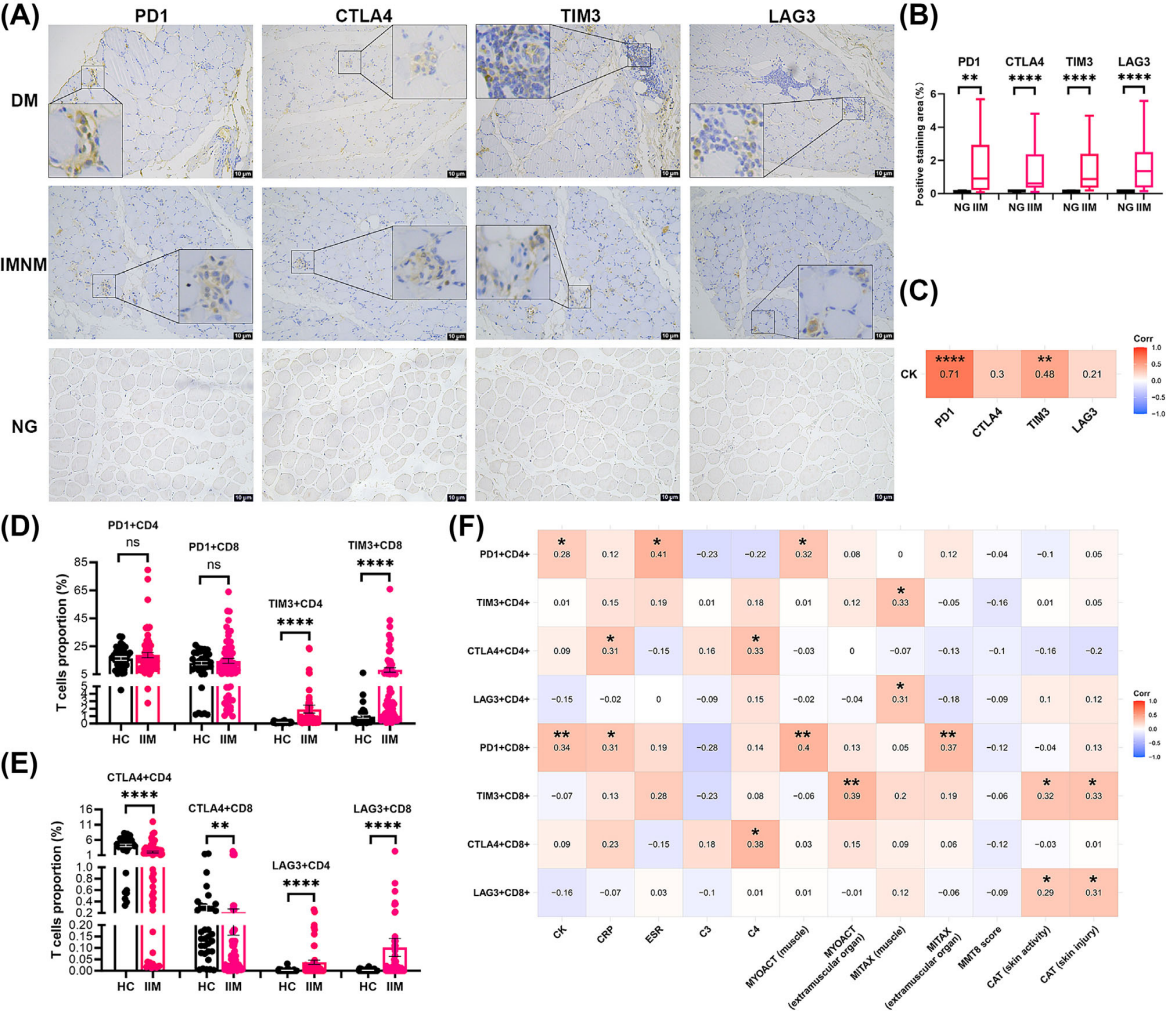
Exhausted T cell increases and is positively correlated with disease activity in patients with IIM. (A, B) Immunohistochemical staining and semi‐quantitatively analysis (area of positive staining) of PD1, CTLA4, TIM3, and LAG3 in skeletal muscle of HC (*n* = 6), patients with DM (*n* = 10) and IMNM (*n* = 21). (C) Correlation analysis between serum CK levels (*n* = 34) and positive staining area of PD1, CTLA4, TIM3, and LAG3 in skeletal muscle of patients with IIM. (D, E) Proportions of exhausted CD4^+^ T cell (PD1^+^CD4^+^ T‐cells, TIM3^+^CD4^+^ T‐cells, CTLA4^+^CD4^+^ T‐cells, and LAG3^+^CD4^+^ T‐cells) and exhausted CD8^+^ T cell (PD1^+^CD8^+^ T‐cells, TIM3^+^CD8^+^ T‐cells, CTLA4^+^CD8^+^ T‐cells, and LAG3^+^CD8^+^ T‐cells) in peripheral blood of HC (*n* = 30) and patients with IIM (*n* = 63). (F) Correlation analysis of exhausted CD4^+^ T cell and exhausted CD8^+^ T cell with clinical indicators and myositis scores. The numbers in the coloured squares are the values of the correlation coefficient (*r)*. CAT: cutaneous assessment tool; CTLA4: cytotoxic T‐lymphocyte‐associated protein‐4; DM: dermatomyositis; HC: healthy control; IIM: idiopathic inflammatory myopathy; INMN: immune‐mediated necrotizing myopathy; LAG3: lymphocyte‐activation gene‐3; MITAX: myositis intention to treat activity index; MMT8: manual muscle testing 8; MYOACT: myositis disease activity assessment visual analogue scale; NG: normal group; PD1: programmed cell death protein‐1; TIM3: T‐cell immunoglobulin and mucin‐domain containing‐3. Scale bar = 10 μm; vs. NG or HC, **P < 0.01, ****P < 0.0001.

The expression of T cell exhaustion‐related genes was increased in the skeletal muscles of patients with DM, JDM, PM, and IMNM compared with NG in public datasets (Figure S7). Immunohistochemical staining also showed elevated expression of PD1 (*P* = 0.0023), CTLA4 (*P* < 0.0001), LAG3 (*P* < 0.0001), and TIM3 (*P* < 0.0001) in the skeletal muscles of patients with IIM, primarily in infiltrating inflammatory cells than in NG (Figure 4A,B). Moreover, a positive correlation was found between CK levels and positive staining areas of PD1 (*r* = 0.7072, *P* < 0.0001), TIM3 (*r* = 0.4808, *P* = 0.0046), but not CTLA4 (*r* = 0.3041, *P* = 0.0757) and LAG3 (*r* = 0.2075, *P* = 0.2545) (Figure 4C). Flow cytometry revealed higher proportions of TIM3^+^CD4^+^ T cells [0.1550% (95% CI: 0.1100, 0.2100) vs. 0.6600% (95% CI: 0.3900, 0.9600), *P* < 0.0001], TIM3^+^CD8^+^ T cells [0.4000% (95% CI: 0.3100, 0.7400) vs. 2.8900% (95% CI: 2.0100, 5.0800), *P* < 0.0001], LAG3^+^CD4^+^ T cells [0.0000% (95% CI: 0.0000, 0.0004) vs. 0.0052% (95% CI: 0.0011, 0.0140), *P* < 0.0001] and LAG3^+^CD8^+^ T cells [0.0000% (95% CI: 0.0000, 0.0000) vs. 0.0073% (95% CI: 0.0017, 0.0180), *P* < 0.0001], but lower proportions of CTLA4^+^CD4^+^ T cells [4.2650% (95% CI: 3.3700, 5.6500) vs. 1.5300% (95% CI: 0.8300, 2.0000), *P* < 0.0001] and CTLA4^+^CD8^+^ T cells [0.1550% (95% CI: 0.1100, 0.2700) vs. 0.0555% (95% CI: 0.0270, 0.1100), *P* = 0.0025] in patients with IIM than in HCs. However, there was no significant change in exhausted PD1^+^CD4^+^ T cells [16.25% (95% CI: 11.80, 20.30) vs. 14.90% (95% CI: 12.00, 16.50), *P* = 0.8878] and PD1^+^CD8^+^ T cells [13.40% (95% CI: 8.82, 18.70) vs. 9.12% (95% CI: 6.19, 13.10), *P* = 0.6639] (Figure 4D,E and Figure S8). We found that exhausted CD4^+^ T cells were associated with CK (PD1^+^CD4^+^, *r* = 0.2833, *P* = 0.0283), CRP (CTLA4^+^CD4^+^, *r* = 0.3144, *P* = 0.0354), ESR (PD1^+^CD4^+^, *r* = 0.4108, *P* = 0.0142), myositis disease activity assessment visual analogue scale of muscle (MAYOACT) (PD1^+^CD4^+^, *r* = 0.3243, *P* = 0.0115), and myositis intention to treat activity index of muscle (MITAX) (TIM3^+^CD4^+^, *r* = 0.3294 *P* = 0.0102; LAG3^+^CD4^+^, *r* = 0.3066 *P* = 0.0172). Exhausted CD8^+^ T cells were associated with CK (PD1^+^CD8^+^, *r* = 0.3369, *P* = 0.0085), CRP (PD1^+^CD8^+^, *r* = 0.3143, *P* = 0.0355), MAYOACT of muscle (PD1^+^CD8^+^, *r* = 0.3959, *P* = 0.0017), extramuscular organ (TIM3^+^CD8^+^, *r* = 0.3933, *P* = 0.0019), MITAX of extramuscular organ (PD1^+^CD8^+^, *r* = 0.3746, *P* = 0.0032), cutaneous assessment tool (CAT) of skin activity (TIM3^+^CD8^+^, *r* = 0.3231, *P* = 0.0118; LAG3^+^CD8^+^, *r* = 0.2863, *P* = 0.0266), and CAT of skin injury (TIM3^+^CD8^+^, *r* = 0.3270, *P* = 0.0108; LAG3^+^CD8^+^, *r* = 0.3072, *P* = 0.0170) (Figure 4F). However, CTLA4^+^CD4^+^ (*r* = 0.3284, *P* = 0.0472) and CTLA4^+^CD8^+^ (*r* = 0.3841, *P* = 0.0173) T cells were positively correlated with C4 levels (Figure 4F).

### Increased exhausted Tregs in the affected skeletal muscle in patients with IIM

**Figure 5:**
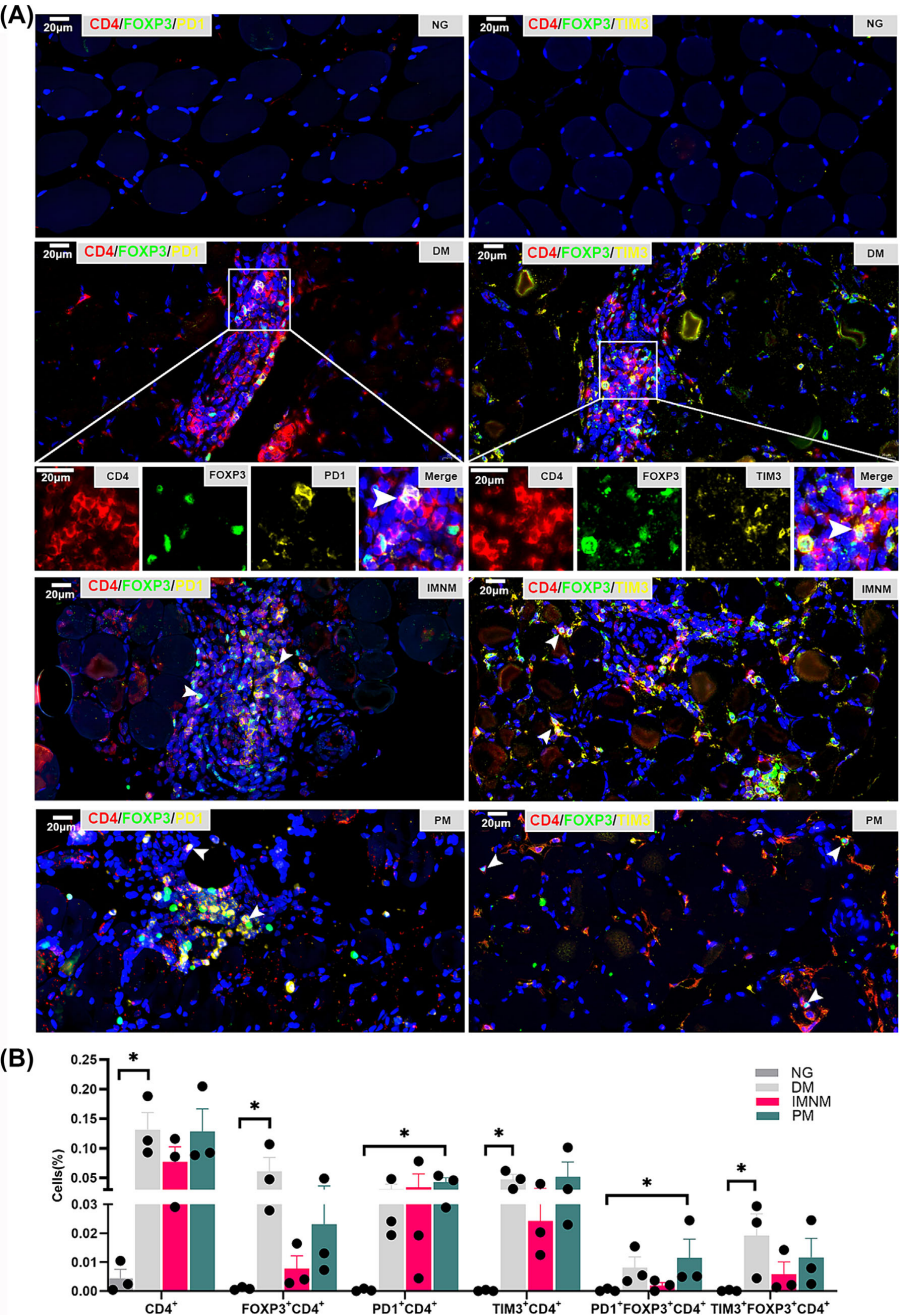
Exhausted Tregs in the affected skeletal muscle in patients with IIM. Multiple immunohistochemical staining (A) for PD1^+^Tregs (PD1^+^FOXP3^+^CD4^+^) and TIM3^+^Tregs (TIM3^+^FOXP3^+^CD4^+^) and semi‐quantitative analysis (B) of CD4^+^, FOXP3^+^CD4^+^, PD1^+^CD4^+^, TIM3^+^CD4^+^, PD1^+^FOXP3^+^CD4^+^, and TIM3^+^FOXP3^+^CD4^+^ cells in skeletal muscle of the NG and patients with DM, PM, and IMNM (*n* = 3). DM: dermatomyositis; IMNM: immune‐mediated necrotizing myopathy; NG: normal group; PD1: programmed cell death protein‐1; PM: polymyositis; TIM3: T‐cell immunoglobulin and mucin‐domain containing‐3. CD4 in red, FOXP3 in green, PD1 and TIM3 in yellow; the merged part is bright orange, scale bar = 20 μm. vs. NG, *P < 0.05.

Multicolour immunohistochemical staining showed increased CD4^+^, FOXP3^+^CD4^+^, TIM3^+^CD4^+^ cells and exhausted Tregs (TIM3^+^FOXP3^+^CD4^+^ cells) in the skeletal muscles of patients with DM (all *P* < 0.05), and increased PD1^+^CD4^+^ cells and exhausted Tregs (PD1^+^FOXP3^+^CD4^+^ cells) in patients with PM (all *P* < 0.05) compared with the NG (Figure 5A,B).

### Adenosine A2B receptor agonist inhibits splenic Treg exhaustion in vivo and in vitro

**Figure 6:**
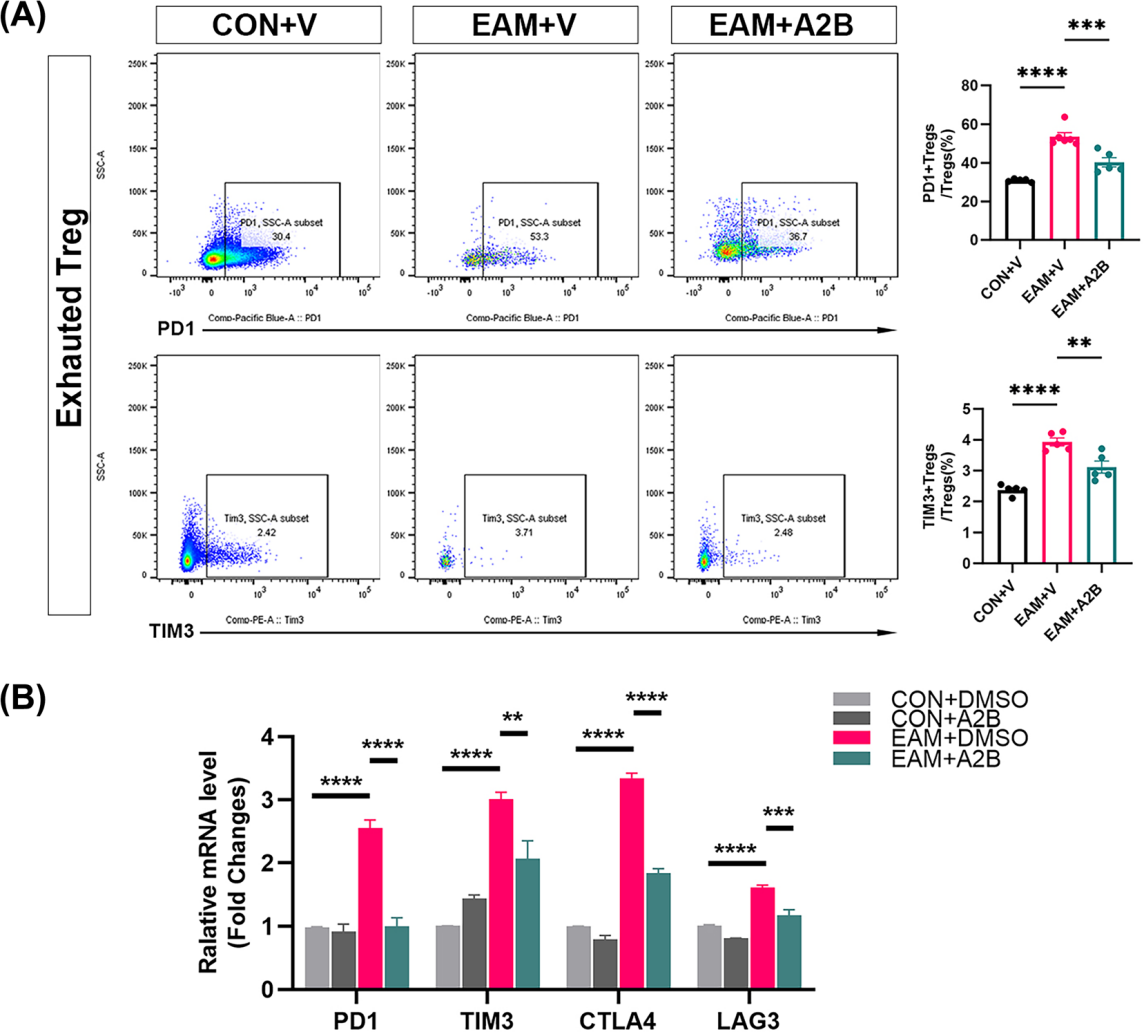
Activation of adenosine A2B receptors inhibits Tregs exhaustion in vivo and in vitro. (A) Flow scatter plots and proportions of exhausted Tregs (splenic PD1^+^ Tregs and TIM3^+^ Tregs) in mice (*n* = 5–6). (B) mRNA levels of PD1, TIM3, CTLA4, and LAG3 in splenic Tregs stimulated with DMSO or adenosine A2B receptor agonist (*n* = 3). CON: control; CTLA4: Cytotoxic T‐lymphocyte‐associated protein‐4; EAM: experimental autoimmune myositis; LAG3: lymphocyte‐activation gene‐3; PD1: programmed cell death protein‐1; TIM3: T‐cell immunoglobulin and mucin‐domain containing‐3;V: vehicle. vs. EAM + V group or EAM + DMSO group, **P < 0.01, ***P < 0.001; ****P < 0.0001.

We found a significant increase in the proportions of splenic PD1^+^ Tregs (30.88% vs. 53.55%, *P* < 0.0001) and TIM3^+^ Tregs (2.37% vs. 3.93%, *P* < 0.0001) in EAM mice compared with the controls. After adenosine A2B receptor agonist intervention, there was a significant decrease in the proportions of splenic PD1^+^Tregs (53.55% vs. 40.28%, *P* = 0.0005) and TIM3^+^Tregs (3.93% vs. 3.11%, *P* = 0.0029) in EAM mice (Figure 6A).

Splenic Tregs from control and EAM mice were stimulated with the adenosine A2B receptor agonist or DMSO in vitro. The mRNA levels of PD1, TIM3, CTLA4, and LAG3 significantly increased in model Tregs (EAM + DMSO) compared with the controls (CON+DMSO) (all *P* < 0.0001), which were significantly decreased (PD1, *P* < 0.0001; TIM3, *P* = 0.0063; CTLA4, *P* < 0.0001; LAG3, *P* = 0.0007) after the adenosine A2B receptor agonist stimulation (Figure 6B).

### Adenosine A2B receptor agonist inhibits HIF‐1α expression

**Figure 7:**
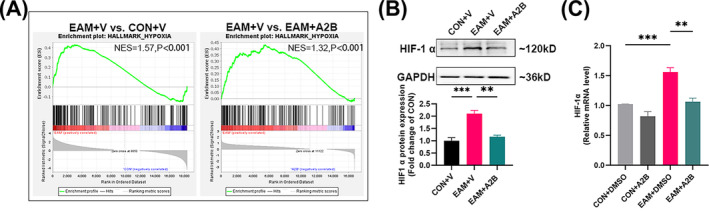
Activation of adenosine A2B receptor inhibits HIF‐1α expression in vivo and in vitro. (A) GSEA of the hypoxia signalling pathway in skeletal muscle of mice. (B) Representative immunoblot bands and grey value analysis of HIF‐1α in mouse skeletal muscle (*n* = 5–6). (C) mRNA levels of HIF‐1α of splenic Tregs stimulated with DMSO or adenosine A2B receptor agonist (*n* = 3). CON: control; EAM: experimental autoimmune myositis; GSEA: gene set enrichment analysis; LW6: an inhibitor of HIF‐1α; NES: normalized enrichment score; V: vehicle. vs. EAM + V or EAM + DMSO group, **P < 0.01, ***P < 0.001.

GSEA revealed significant upregulation of the hypoxia signalling pathway in both patients with PM and DM (Figure S5E‐H) compared with the NG and in EAM mice (CON+V vs. EAM+V, *P* < 0.001, Figure 7A) compared with controls. This pathway was significantly downregulated following the adenosine A2B receptor agonist intervention (EAM+V vs. EAM + A2B, *P* < 0.001, Figure 7A).

In vivo, the elevated HIF‐1α protein levels (*P* = 0.0008) in the skeletal muscle of EAM mice compared to controlssignificantly decreased following the adenosine A2B receptor agonist intervention (*P* = 0.0019, Figure 7B). In vitro, the model (EAM + DMSO) splenic Tregs exhibited a significant increase in HIF‐1α mRNA levels (*P* = 0.0007) compared with the controls (CON+DMSO), which were also significantly decreased after the adenosine A2B receptor agonist stimulation (*P* = 0.0012, Figure 7C).

### HIF‐1α inhibition downregulates p‐STAT3^+^Th17, upregulates Tregs, and suppresses Treg exhaustion, thereby slowing down the progression of EAM

**Figure 8:**
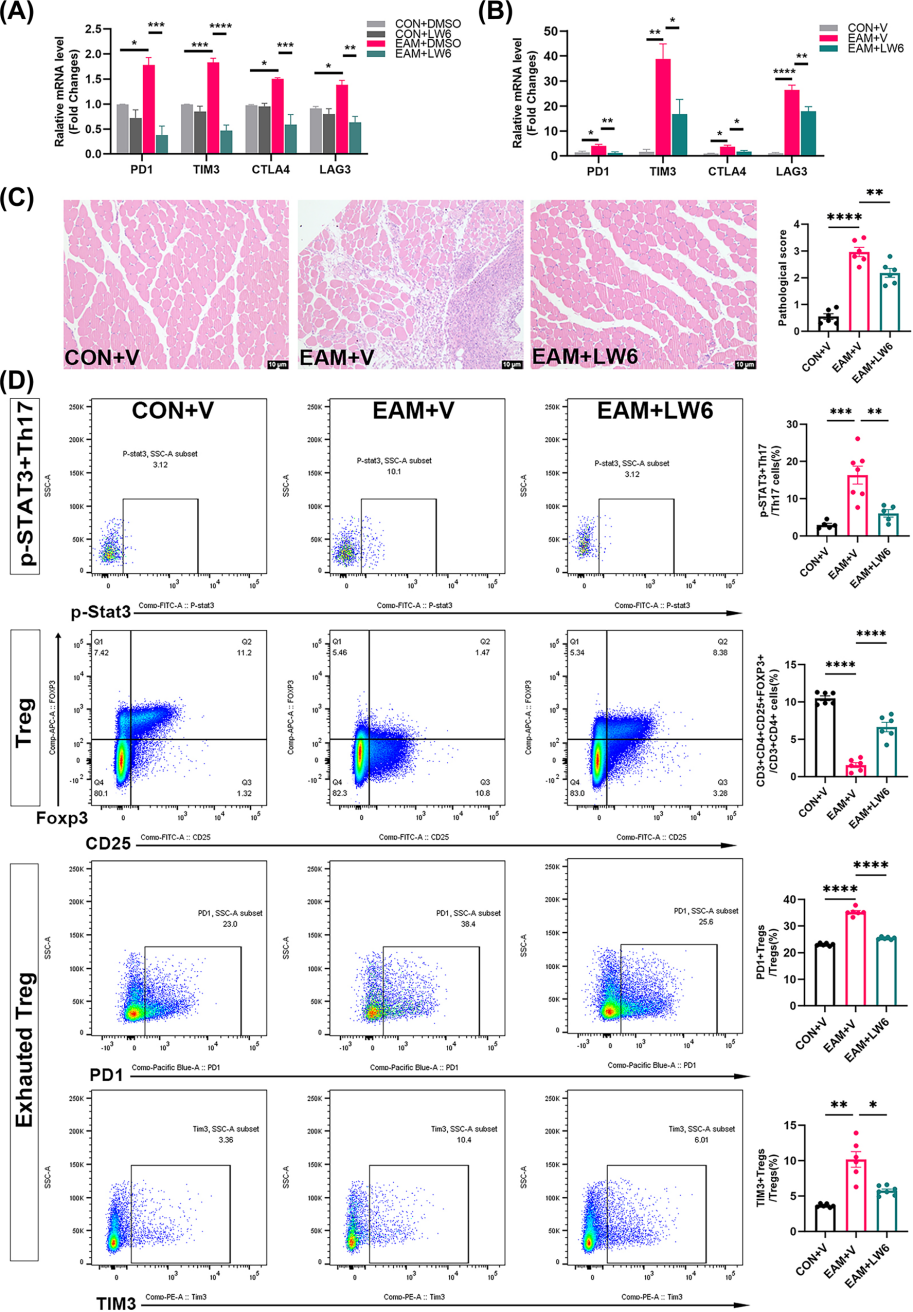
HIF‐1α inhibition slows down the progression of EAM by upregulating Tregs, downregulating p‐STAT3^+^Th17, and suppressing exhausted Tregs. (A) mRNA levels of PD1, TIM3, CTLA4, and LAG3 of splenic Tregs stimulated with DMSO or LW6 (*n* = 3). (B) mRNA levels of PD1, TIM3, CTLA4, and LAG3 in mouse skeletal muscle (*n* = 6). (C). Hematoxylin and Eosin staining and pathological damage and inflammation scores of mouse skeletal muscle (*n* = 6). (D) Flow scatter plots and proportions of mouse splenic p‐STAT3^+^Th17, Tregs, and exhausted Tregs (splenic PD1^+^Tregs and TIM3^+^Tregs). CON: control; EAM: experimental autoimmune myositis; LW6: an inhibitor of HIF‐1α; PD1: programmed cell death protein‐1; TIM3: T‐cell immunoglobulin and mucin‐domain containing‐3; V: vehicle. Scale bar = 10 μm; vs. EAM + DMSO or EAM + V group, *P < 0.05, **P < 0.01, ***P < 0.001, ****P < 0.0001.

The sorted model Tregs (EAM + DMSO) exhibited significantly elevated PD1 (*P* = 0.0121), TIM3 (*P* = 0.0004), CTLA4 (*P* = 0.0187), and LAG3 (*P* = 0.0192) mRNA levels compared with the controls (CON+DMSO), which significantly decreased after LW6 stimulation (*P* = 0.0003, *P* < 0.0001,*P* = 0.0007, and *P* = 0.0012, respectively; Figure 8A). PD1 (*P* = 0.0127), TIM3 (*P* = 0.0037), CTLA4 (*P* = 0.0229), and LAG3 (*P* < 0.0001) mRNA levels were higher in skeletal muscle of EAM mice compared with the controls, which decreased after LW6 intervention (*P* = 0.0086, *P* = 0.0338, *P* = 0.0417, and *P* = 0.0066, respectively; Figure 8B).

The affected skeletal muscles of EAM mice exhibited worsened muscle damage and inflammatory infiltration and higher pathological injury scores than the controls (*P* < 0.0001). Nevertheless, EAM mice showed a decrease in the pathological injury score (*P* = 0.0044) after LW6 intervention (Figure 8C). EAM mice had significantly reduced body weight (20.29 vs. 18.40 g, *P* = 0.0007), increased spleen weight (0.11 vs. 0.38 g, *P* = 0.0012), and decreased muscle strength (159.30 vs. 110.70 g, *P* = 0.0004), compared with the control mice. Following LW6 intervention, EAM mice exhibited an increase in body weight (18.40 vs. 19.81 g, *P* = 0.0072), a significant decrease in spleen weight (0.38 vs. 0.25 g, *P* = 0.0294), and partial recovery in muscle strength (110.70 vs. 141.60 g, *P* = 0.0133) (Figure S9A–C). Additionally, EAM mice showed elevated serum levels of IL‐1β (3.93 vs. 8.72 pg/mL, *P* = 0.0004), IL‐6 (3.75 vs. 38.43 pg/mL, *P* < 0.0001), and TNF‐α (8.70 vs. 22.51 pg/mL, *P* < 0.0001) compared with the controls. After LW6 intervention, IL‐1β (8.72 vs. 4.88 pg/mL, *P* = 0.0019) and IL‐6 (38.43 vs. 21.95 pg/mL, *P* = 0.0007) levels decreased in EAM mice (Figure S9D–F).

After LW6 intervention, the increased splenic Th17 cell proportion (0.24% vs. 0.72%, *P* < 0.0001) and p‐STAT3^+^Th17 (3.0% vs. 16.32%, *P* = 0.0003) compared with controls significantly decreased (Th17, 0.72% vs. 0.40%, *P* < 0.0001, Figure S10; p‐STAT3^+^Th17, 16.32% vs. 6.06%, *P* = 0.0025, Figure 8D), and the decrease Tregs proportion (10.50% vs. 1.56%, *P* < 0.0001) compared with controls significantly increased (1.56% vs. 6.65%, *P* < 0.0001, Figure 8D). Additionally, EAM mice showed an elevated Th1 cell proportion (0.03% vs. 0.36%, *P* = 0.0009) compared with the controls, but Th1 and Th2 cell proportions did not change after LW6 intervention (Figure S10). Furthermore, the proportions of splenic PD1^+^Tregs (23.00% vs. 35.23%, *P* < 0.0001) and TIM3^+^Tregs (3.65% vs. 10.17%, *P* = 0.0036) significantly increased in the EAM mice compared with the controls. Following LW6 intervention, splenic PD1^+^Tregs (35.23% vs. 25.38%, *P* < 0.0001) and TIM3^+^Tregs (10.17% vs. 5.75%, *P* = 0.0203) proportions significantly decreased in EAM mice (Figure 8D).

## Discussion

CD73 is pivotal for producing extracellular adenosine, influencing immune regulation and inflammation through adenosine nucleotide metabolism in rheumatic autoimmune diseases.^26^ In this study, we observed heightened CD73 expression, particularly in infiltrating inflammatory cells within affected muscles in IIM subtypes. Given the minimal presence of inflammatory cells in normal skeletal muscle, the increased CD73 levels in inflammatory cells may be a compensatory mechanism to enhance their anti‐inflammatory properties. Additionally, we found that ADA is upregulated in the skeletal muscles, and the elevated serum ADA level in patients with IIM was positively correlated with the levels of the enzymatic profile of muscles. Previous studies have identified increased serum levels of ADA in autoimmune or inflammatory conditions.^27^, ^28^ In cases of inflammation, ADA facilitates the degradation of adenosine, consequently inhibiting the activation of adenosine A2B receptors,^28^ suggesting that elevated ADA may contribute to disease progression in IIM by excessive adenosine degradation. In vivo, adenosine A2B receptor agonist is the most effective in mitigating skeletal muscle damage and inflammation and enhancing muscle strength, which aligns with Gnad's study, where A2B adenosine receptor activation notably increased muscle mass and strength.^29^ This finding lays a foundation for future drug development targeting the adenosine A2B receptor to benefit a wide range of patients. However, the mechanism by which adenosine A2B receptor activation restores muscle strength in EAM requires further investigation.

T cell exhaustion is observed in the skeletal muscles of patients with IMNM and sporadic inclusion body myositis,^14^ which is consistent with our observation of increased expression of T‐cell exhaustion genes in skeletal muscles of IIM subtype. CD8^+^ T cells from anti‐MDA5 antibody‐positive DM patients have shown significantly higher levels of LAG‐3, TIM‐3, and PD‐1 than anti‐MDA5 antibody‐negative IIM patients and HCs.^30^ Although we did not classify patients with IIM based on specific myositis antibody subtypes to compare levels of exhausted T cells (owing to limited sample size), we observed increased expression of LAG‐3 and TIM‐3 in both CD4^+^ and CD8^+^ T cells in IIM. Additionally, correlation analysis revealed a positive association between TIM3^+^ or LAG3^+^CD8^+^ T cells and CAT scores. Another study indicated that peripheral PD1^+^CD8^+^ T cells, which express elevated cytolytic molecules, constitute a pathogenic cell subset in IIM,^16^ and IFN‐γ‐induced PD‐L1 expression on myotubes has a protective effect in a myositis model.^16^ Our study findings revealed that increased expression of T cell exhaustion‐related genes in skeletal muscles was associated with serum CK levels. Exhausted T cells, such as PD1^+^CD8^+^ T cells or PD1^+^CD4^+^ T cells, showed a positive correlation with serum CK levels and MYOACT scores. These results suggest that exhausted T cells may not indicate a suppressed autoimmune response as previously reported,^31^ but rather a potential pathogenic role. Further investigation into alterations in other functional molecules of exhausted T cells is recommended to better understand their involvement in IIM. Notably, we found that the peripheral CTLA4^+^ T cell proportions in IIM were lower than those in controls and were positively correlated with serum C4 levels. Similarly, the low expression of CTLA4 in Foxp3^+^CD25^+^CD4^+^ Tregs has been negatively associated with disease activity in patients with systemic lupus erythematosus.^32^ These findings suggest that the role of classical immune checkpoint proteins may not always be consistent and clinicians should consider the functional status of T cells in clinical practice.

The adenosine system has been reported to regulate T‐cell exhaustion.^10^, ^33^, ^34^ We demonstrated that the adenosine A2B receptor activation suppressed the expression of exhaustion‐related genes in skeletal muscles, reducing the proportions of exhausted splenic CD4^+^ T cells while regulating the Th17/Treg balance. However, in vitro, the suppressed exhausted CD4^+^ T cells did not exhibit the expected increase in cytokine secretion but decreased instead. This may attribute to the complex scenario resulting from the distinct effects of the adenosine A2B agonist on various CD4^+^ T cell subtypes. Investigating the impact of A2B adenosine receptor agonists on exhausted CD4^+^ T cell subtypes is necessary. A study employing single‐cell sequencing revealed Treg cell exhaustion in patients with severe systemic lupus erythematosus,^35^ and we confirmed exhausted Tregs in the skeletal muscles of patients with IIM via staining. Furthermore, adenosine A2B receptor activation inhibited splenic Treg exhaustion in vivo and in vitro.

Combining bioinformatics analysis combined with the results of the cell intervention experiments, we found that activation of the adenosine A2B signalling pathway may inhibit the STAT3 signalling pathway by down‐regulating HIF‐1α and inhibiting Th17 differentiation, and thereby improving inflammation levels in EAM mice. Activation of the STAT3 signalling pathway can regulate the expression of transcription factors required for Th17 cell differentiation and promote Th17 differentiation.^36^ A previous study revealed that under hypoxic conditions, HIF‐1α can promote Th17 cell differentiation and inhibit the generation of Tregs.^37^ Additionally, there is a close regulation and synergistic effect between HIF‐1α and STAT3 in inflammatory injury.^38^ Therefore, targeting Th17 differentiation against A2B/HIF‐1α/STAT3 signalling may reduce inflammation in EAM. Different perspectives exist regarding the regulatory role of HIF‐1α in T cell exhaustion.^39^, ^40^ Our study revealed that inhibiting HIF‐1α can suppress splenic Treg exhaustion. Therefore, activating adenosine A2B receptors may regulate the Th17/Treg balance and inhibit Treg exhaustion by suppressing HIF‐1α expression. For researchers, this finding broadens the spectrum of diseases in which cross talk exists between immunity and metabolism.

However, our study had some limitations. First, the sample size was relatively small; therefore, a larger sample size is needed to gain a better understanding of the exhaustion characteristics of T cells, especially CD4^+^ T cell subtypes, in different IIM subtypes. Secondly, we did not use knockout mice to further confirm the critical role of the adenosine A2B receptor of Th17 and Tregs in EAM. These limitations provide directions for future research.

In conclusion, we found that exhausted T cells may be pathogenic in IIM, and adenosine A2B receptor activation mitigates EAM progression by downregulating HIF‐1α, regulating the Th17/Treg balance, and inhibiting Treg exhaustion. These findings provide new insights into the pathogenesis of IIM and offer potentially novel therapeutic targets for its treatment.

## Conflict of interest

The authors have declared no conflicts of interest.

## Funding

This study was supported by Sichuan Science and Technology Program (grant numbers: 2021JDRC0045, 2021YFS0164 and 24ZDYF0224), the Clinical Research Incubation Project of West China Hospital, Sichuan University (grant numbers: 2019HXFH038 and 2021HXFH018), and the 1.3.5 Project for Disciplines of Excellence, West China Hospital, Sichuan University (grant number: ZYJC21024).

## Supporting information


**Data S1.** Supplementary Tables S1–S6.

**Data S2.** Supplementary materials and methods.

**Data S3.** Supplementary Figure S1–S10.

## Data Availability

The data that support the findings of this study are available from the corresponding author upon reasonable request.
