# Supplementary material for: Adenosine A2B receptor activation regulates the balance between T helper 17 cells and regulatory T cells, and inhibits regulatory T cells exhaustion in experimental autoimmune myositis
Source: J Cachexia Sarcopenia Muscle. 2024 Sep 16;15(6):2460–75. doi: 10.1002/jcsm.13581 (PMC11634480; doi:10.1002/jcsm.13581)
Supplement: Supplementary file 1 — Data S1. Supplementary Tables S1–S6. [file JCSM-15-2460-s002.docx]

**Supplementary Material Table S1. Clinical data of healthy controls and patients with IIM for serological detection**

| **Items** | **Patients with IIM** | **Controls** | **P-value** |
| --- | --- | --- | --- |
| **Females** | 44/64 (69%) | 25/35 (71%) | P>0.05 |
| **Age (years)** | 49±9 | 44±11 | P>0.05 |
| **Subtypes of IIM** |  |  |  |
| DM | 43 (68%) |  |  |
| PM | 8 (12%) |  |  |
| IMNM | 13 (20%) |  |  |
| **Myositis-specific antibodies** |  |  |  |
| Anti-MDA5 | 24 (37.5%) |  |  |
| Anti-SRP | 8 (12.5%) |  |  |
| Anti-HMGCR | 2 (3.1%) |  |  |
| Anti-Mi-2 | 1 (1.6%) |  |  |
| Anti-SAE | 1 (1.6%) |  |  |
| Anti-Jo-1 | 2 (3.1%) |  |  |
| Anti-EJ | 1 (1.6%) |  |  |
| Anti-PL-7 | 1 (1.6%) |  |  |
| Anti-PL-12 | 1 (1.6%) |  |  |
| MSA-negative | 23 (35.9%) |  |  |
| **Laboratory data** |  |  |  |
| CK, IU/L | 160 (50, 2012) |  |  |
| ALT, IU/L | 50 (25, 106) |  |  |
| AST, IU/L | 40 (22, 95) |  |  |
| LDH, IU/L | 296 (195, 520) |  |  |
| HBDH, IU/L | 226 (159, 397) |  |  |
| Myoglobin, ng/mL | 61 (24, 880) |  |  |
| CRP, mg/L | 4.5 (2.1, 15.5) |  |  |
| ESR, mm/h | 38.5 (19.5, 65.3) |  |  |
| C3, g/L | 0.931 (0.745, 0.993) |  |  |
| C4, g/L | 0.251 (0.197, 0.310) |  |  |

Apart from age represented as mean ± standard deviation, other data is expressed as N (%) or median (25th percentile, 75th percentile). IIM: Idiopathic inflammatory myopathy; DM: Dermatomyositis; PM: Polymyositis; IMNM: Immune-mediated necrotizing myopathy; MDA5: melanoma differentiation-associated gene 5; SRP: signal recognition particle; HMGCR: hydroxymethylglutaryl-CoA reductase; SAE: small ubiquitin-like modifier activating enzyme; MSA: myositis-specific autoantibody. ALT : alanine aminotransferase; AST: aspartate aminotransferase; CK: creatine kinase; LDH: lactate dehydrogenase; HBDH: hydroxybutyrate dehydrogenase.

**Supplementary Material Table S2. Clinical data of healthy controls and patients with IIM for flow cytometry detection**

| **Items** | **IIM** | **HC** | **P-value** |
| --- | --- | --- | --- |
| **Females** | 44/63 (70%) | 17/30 (57%) | P>0.05 |
| **Age (years)** | 47±11 | 50±11 | P>0.05 |
| **Subtypes of IIM** |  |  |  |
| DM | 26 (41.3%) |  |  |
| IMNM | 14 (22.2%) |  |  |
| ASS | 16 (25.4%) |  |  |
| Unclassified IIM | 7 (11.1%) |  |  |
| **Myositis-specific antibodies** |  |  |  |
| Anti-MDA5 | 15 (24%) |  |  |
| Anti-SAE | 1 (1.6%) |  |  |
| Anti-Jo-1 | 13 (21%) |  |  |
| MSA-negative | 8 (13%) |  |  |
| Anti-Mi-2 | 1 (1.6%) |  |  |
| Anti-TIF-1γ | 1 (1.6%) |  |  |
| Anti-NXP2 | 3 (5%) |  |  |
| Anti-HMGCR | 8 (13%) |  |  |
| Anti-SRP | 6 (10%) |  |  |
| Anti-PL-12 | 2 (3%) |  |  |
| Anti-PL-7 | 2 (3%) |  |  |
| Anti-PM-SCL | 1 (1.6%) |  |  |
| Anti-EJ | 1 (1.6%) |  |  |
| **Laboratory data** |  |  |  |
| CK (IU/L) | 148 (56, 981) |  |  |
| ALT (IU/L) | 35 (17, 88) |  |  |
| AST (IU/L) | 31 (18, 75 ) |  |  |
| LDH (IU/L) | 323 (246, 440) |  |  |
| HLDH (IU/L) | 239 (191, 315) |  |  |
| CRP(mg/L) | 5.7 (3.6, 12.9) |  |  |
| ESR (mm/h) | 29.0 (11.0, 50.0) |  |  |
| C3 (g/L) | 0.8805 (0.7060, 0.9885) |  |  |
| C4 (g/L) | 0.2005 (0.1618, 0.2548) |  |  |
| **Myositis activity score** |  |  |  |
| MYOACT of muscle | 3.7 (1.8, 7.0) |  |  |
| MYOACT of extramuscular organ | 2.5 (1.3, 3.7) |  |  |
| MITAX of muscle | 1.0 (0.0, 3.0) |  |  |
| MITAX of extramuscular organs | 1.7 (0.5, 2.2) |  |  |
| CAT of skin activity | 1.0 (0.0, 3.0) |  |  |
| CAT of skin injury | 0.0 (0.0, 2.0) |  |  |
| MMT8 | 135 (98, 150) |  |  |

Apart from age represented as mean ± standard deviation, other data is expressed as N (%) or median (25th percentile, 75th percentile). HC: Healthy control; IIM: Idiopathic inflammatory myopathy; DM: Dermatomyositis; ASS: anti-synthetase syndrome; IMNM: Immune-mediated necrotizing myopathy; MDA5: melanoma differentiation-associated gene 5; SAE: small ubiquitin-like modifier activating enzyme; Jo-1: histidyl-tRNA synthetase; MSA: myositis-specific autoantibody; TIF-1γ: transcription intermediary factor-1γ; NXP2: nuclear matrix protein 2; HMGCR: hydroxymethylglutaryl-CoA reductase; SRP: signal recognition particle; PL: threonyl-tRNA synthetase; EJ: aminoacyl-tRNA synthetase; CK: Creatine kinase; LDH: Lactate dehydrogenase; HBDH: Hydroxybutyrate dehydrogenase; ALT: Alaninetransaminase; AST: Aspartate transaminase; MYOACT: Myositis disease activity assessment visual analogue scale; MITAX: Myositis intention to treat activity index; CAT: Cutaneous assessment tool; MMT8: Manual muscle testing 8. SLEDAI-2K: SLE disease activity index 2000. vs. Healthy controls. *p <0.05.

**Supplementary Material Table S3: Clinical data of normal controls and patients with IIM for skeletal muscle detection**

| **Items** | **Patients with IIM** | **Controls (patients with osteoarthritis)** | **P-value** |
| --- | --- | --- | --- |
| **Females** | 24/34 (71%) | 4/6 (67%) | P>0.05 |
| **Age (years)** | 52±13 | 63±6 | P>0.05 |
| **Subtypes of IIM** |  |  |  |
| DM | 10 (29%) |  |  |
| PM | 3 (9%) |  |  |
| IMNM | 21 (62%) |  |  |
| **Duration of disease (months)** | 16 (4-24) |  |  |
| **Myositis-specific antibodies** |  |  |  |
| Anti-MDA5 | 3 (9%) |  |  |
| Anti-SRP | 12 (35%) |  |  |
| Anti-HMGCR | 5 (15%) |  |  |
| Anti-Mi-2 | 1 (3%) |  |  |
| Anti-NXP2 | 1 (3%) |  |  |
| MSA-negative | 12 (35%) |  |  |
| **Laboratory data** |  |  |  |
| CK, IU/L | 1747 (171-3443) |  |  |
| ALT, IU/L | 97 (46-202) |  |  |
| AST, IU/L | 95 (53-223) |  |  |
| LDH, IU/L | 426 (261-786) |  |  |
| HBDH, IU/L | 322 (185-618) |  |  |
| CRP, mg/L | 4.3 (1.8-10.6) |  |  |
| ESR, mm/h | 39 (21-61) |  |  |
| C3, g/L | 0.839 (0.692-0.990) |  |  |
| C4, g/L | 0.192 (0.141-0.292) |  |  |

Apart from age represented as mean ± standard deviation, other data is expressed as N (%) or median (25th percentile, 75th percentile). IM: Idiopathic inflammatory myopathy; DM: Dermatomyositis; PM: Polymyositis; IMNM: Immune-mediated necrotizing myopathy; MDA5: melanoma differentiation-associated gene 5; SRP: signal recognition particle; HMGCR: hydroxymethylglutaryl CoA reductase; NXP2：nuclear matrix protein 2; MSA: myositis-specific autoantibody; ALT : alanine aminotransferase; AST: aspartate aminotransferase; CK: creatine kinase; LDH: lactate dehydrogenase; HBDH: hydroxybutyrate dehydrogenase.

**Supplementary Material Table S****4:** **Skeletal muscle damage and inflammation scoring criteria of EAM mice.**

Pathological scoring of skeletal muscle damage and inflammation in mice was conducted as described previously [1, 2]. The evaluation was conducted blindly by assessing HE-stained sections of skeletal muscle under a microscope.

| Grade | Skeletal muscle injury | Score |
| --- | --- | --- |
| I | Involving <5 muscle fibers | 1 |
| Ⅱ | Involving 5-30 muscle fibers | 2 |
| Ⅲ | Involving a bundle of muscle fibers | 3 |
| Ⅳ | Diffuse infiltration of inflammatory cells | 4 |

Add 0.5 point when there are multiple lesions of skeletal muscle tissue.

At 200× magnification, five random H&E-stained fields of view were selected for scoring and the average score was used as the final pathological score for skeletal muscle damage and inflammation.

**Supplementary Material Table S5: Information of IIM datasets.**

| GSE dataset | Sequencing platform | Skeletal muscle specimen | Date |
| --- | --- | --- | --- |
| GSE128470 | GPL96 [HG-U133A] Affymetrix Human Genome U133A Array | 65 patients with IIM (12 DM, 26 IBM, 6 IMNM, 7 PM and 14 non-specific myositis) and 12 controls | 2019 |
| GSE48280 | GPL6244 [HuGene-1_0-st] Affymetrix Human Gene 1.0 ST Array [transcript (gene) version] | 14 patients with IIM (5 DM, 5 PM, 4 IBM）and 12 controls | 2013 |
| GSE39454 | GPL570 [HG-U133_Plus_2] Affymetrix Human Genome U133 Plus 2.0 Array | 31 patients with IIM (5 IMNM, 8 DM, 8 PM and 10 IBM) and 5 controls | 2012 |
| GSE3112 | GPL96 [HG-U133A] Affymetrix Human Genome U133A Array | 29 patients with IIM (23 IBM, 6 PM) and 11 controls | 2005 |
| GSE11971 | GPL96 [HG-U133A] Affymetrix Human Genome U133A Array | 19 patients with IIM and 4 controls | 2008 |
| GSE1551 | GPL96 [HG-U133A] Affymetrix Human Genome U133A Array | 13 patients with IIM and 10 controls | 2004 |
| GSE5370 | GPL96 [HG-U133A] Affymetrix Human Genome U133A Array | 5 patients with DM and 4 controls | 2006 |
| GSE3307 | GPL96 [HG-U133A] Affymetrix Human Genome U133A Array | 21 patients with JDM and 13 controls | 2005 |

IIM: Idiopathic inflammatory myopathy; GSE: Gene Expression Omnibus; DM: Dermatomyositis; PM: Polymyositis; IMNM: Immune-mediated necrotizing myopathy; IBM: Inclusion body myositis.

**Supplementary Material Table S6: Primer sequence.**

| Gene Name | Forward Primer (5'→3') | Reverse Primer(3'→5') |
| --- | --- | --- |
| GAPDH | AGAACATCATCCCTGCATCC | AGTTGCTGTTGAAGTCGC |
| HIF-1α | ACCTTCATCGGAAACTCCAAAG | ACTGTTAGGCTCAGGTGAACT |
| CTLA4 | CATGGTGTCGCCAGCTTTC | GGTAATCTAGGAAGCCCACTGTA |
| PD1 | GCACCCCAAGGCAAAAATCG | CAATACAGGGATACCCACTAGGG |
| TIM3 | ACTGGTGACCCTCCATAATAACA | GCAGTTCTGATCGTTTCTCCA |
| LAG3 | CCTCGATGATTGCTAGTCCCT | GTAGACAGGCACTCGGTTCTG |

[1] T. Kojima, N. Tanuma, Y. Aikawa, T. Shin, A. Sasaki, Y. Matsumoto, Myosin-induced autoimmune polymyositis in the rat, Journal of the neurological sciences 151 (1997) 141-148.

[2] K. Kohyama, Y. Matsumoto, C-protein in the skeletal muscle induces severe autoimmune polymyositis in Lewis rats, Journal of neuroimmunology 98 (1999) 130-135.
