# Supplementary material for: Adenosine A2B receptor activation regulates the balance between T helper 17 cells and regulatory T cells, and inhibits regulatory T cells exhaustion in experimental autoimmune myositis
Source: J Cachexia Sarcopenia Muscle. 2024 Sep 16;15(6):2460–75. doi: 10.1002/jcsm.13581 (PMC11634480; doi:10.1002/jcsm.13581)
Supplement: Supplementary file 2 — Data S2. Supplementary materials and methods. [file JCSM-15-2460-s001.docx]

**1.1** **Diagnostic criteria, exclusion criteria, activity evaluation tools, and measurement homogeneity**

All patients met the EULAR/ACR criteria (2017) for IIM (ICD-11:4A41) [1]. Patients diagnosed with IMNM also met the European Neuromuscular Center (ENMC) criteria (2017) for IMNM [2]. Patients diagnosed with ASS also met the diagnostic criteria proposed by Solomon et al. in 2011 [3]. Following exclusion of IMNM and ASS, patients who met the EULAR/ACR criteria (2017) for PM were diagnosed accordingly. Patients with IIM were diagnosed by eight rheumatologists with over 15 years of clinical experience. All clinical samples were obtained following the standard diagnostic and therapeutic procedures of patients and the voluntary consent of controls.

Patients who were under 18 years of age, pregnant or breastfeeding women, individuals with other autoimmune diseases, tumors, HIV infection, chronic liver disease, or other muscle disorders, as well as patients who engaged in vigorous exercise within one week before recruitment, were excluded from the study.

Myositis Disease Activity Assessment Tool (MDAAT) and Myositis Intention to Treat Activity Index (MITAX) are used to assess disease activity of IIM. Cutaneous Assessment Tool (CAT) is used to assess skin and mucosal damage and activity of IIM. Manual Muscle Testing 8 (MMT8) was used to assess muscle strength of IIM. The reliability and validity of the above 4 scales are good in patients with IIM [4].

We used above standardized activity assessment tools to evaluate patients with IIM, ensuring consistency by assigning the same trained research assistant to assess patients using relevant scales. Furthermore, we maintained consistency by having the same trained attending physician review the data collection and processing processes to identify potential biases or systematic errors. Finally, we implemented standardized operational procedures for sample collection (performed by uniformly trained healthcare personnel), processing, and detection (consistently by the same laboratory technician) of biological specimens to minimize variability introduced by different procedures or operators.

**1.2 EAM model construction**

On the 1st and 7th days, potassium chloride solution with 1.5 mg of guinea pig myosin (saline for Control) and Freund’s complete adjuvant (Sigma-Aldrich) (containing 10mg/ml Mycobacterium tuberculosis; BD Difco) were emulsified and injected subcutaneously into mice’s left hind limb. Immediately after each immunization, 200 μl of saline with 500 ng of pertussis toxin (GLPbio) was injected intraperitoneally. Incomplete Freund’s adjuvant emulsion with 0.375mg of guinea pig myosin (saline for Control) was injected subcutaneously at the base of the tail twice a week to boost the immune response. Mice were used as models 14 days after the initial immunization. In our study, mice were sacrificed on day 14 to isolate splenic T lymphocytes for cell experiments, on day 17 in the CD73 inhibitor intervention experiment, and on day 21 in the four adenosine agonist and HIF-1α inhibitor intervention experiments. Peripheral blood, left gastrocnemius muscle tissue, and spleen samples were collected at these time points.

**1.3 Assessment of muscle strength**

We used a grip strength meter to measure the muscle strength of the mice by gently pulling their tails back until the mice released their grip and recording the maximum grip strength after three consecutive measurements for each mouse.

**1.4 Immunohistochemical staining**

Tissue sections were processed with dewaxing and hydration, followed by microwave heat-induced repair. After blocking with 3% hydrogen peroxide and 5% goat serum, the sections were incubated with primary antibodies (CD73, 1:1000, Aproteintech; ADA, 1:50, Aproteintech; PD1, 1:200, CST; TIM3, 1:100, CST; CTLA4, 1:400, Abcam; LAG3, 1:200, CST). Incubation condition were 60 minutes at 37°C for CD73 and ADA, and overnight at 4°C for PD1, TIM3, CTLA4, and LAG3. Subsequent steps included applying secondary antibodies (goat anti-mouse/rabbit-HRP secondary antibodies; 1:1000, Affinity; 40 minutes at 37°C), DAB staining (zsbio, China). Five fields of view at 200× magnification were randomly selected from each section, and the positive staining area percentage for each index was calculated using ImageJ software. The average was then taken as a semi-quantitative result.

The Four-color Multiplex Immunohistochemistry Staining Kit (Mouse/Rabbit Universal Secondary Antibodies, Absin, China) was used for conducting multiple immunohistochemical staining. The process included deparaffinization, hydration, antigen retrieval, and blocking steps, following the same protocol as single-marker immunohistochemical staining. Tissues were incubated with CD4 primary antibody (1:100, Santa Cruz) for 1 hour at room temperature. Subsequently, HRP-conjugated secondary antibody was applied for 10 minutes, followed by TSA fluorescent stain 650 (1:100 dilution with signal amplification solution) for 10 minutes. After microwave treatment and cooling, staining continued with PD1 (1:100, TSA 570) and FOXP3 (1:100, TSA 520), or TIM3 (1:1000, TSA 570) and FOXP3 (1:100, TSA 520), with overnight primary antibody incubation. Nuclei were counterstained with DAPI, and sections were sealed with an anti-fluorescence quenching sealer upon completion.

Five random fields at 400× magnification were selected per slide, and ImageJ software was used to quantify the number of positively stained cells (single positive, double positive, or triple positive). The average of ratio of positively stained cells to total cells was calculated as a semi-quantitative result.

**1.5 Mice grouping and tissue sampling**

Following one week of adaptive feeding, 24 specific pathogen-free female Balb/c mice (8 weeks old, weighing 19 ± 1g) were divided into four groups (n=6): Control + Saline, Control + CD73 inhibitor, EAM + Saline, and EAM + CD73 inhibitor. Additionally, 36 female Balb/c mice were randomly allocated into six groups (n=6): Control + Vehicle, EAM + Vehicle, EAM + adenosine A1 or A2A or A2B or A3 receptor agonist. Furthermore, 24 female Balb/c mice were randomly assigned to four groups (n=6): Control + Vehicle, Control + HIF-1α inhibitor, EAM + Vehicle, and EAM + HIF-1α inhibitor. Muscle strength and body weight were evaluated before and after modeling. Anesthesia was induced using 1% pentobarbital sodium intraperitoneally. Blood samples were obtained from the eye for analysis. Spleens were weighed and stored for flow cytometry analysis. The gastrocnemius muscle from the left hind limb was dissected, with one half fixed and the other half frozen for future use.

**1.6 Splenic T lymphocyte sorting**

*Single-cell suspension preparation*

Fresh spleens from Balb/c mice were mechanically dissociated through a 70μm screen using a 1mL syringe plunger. The cells were then washed with a PBS solution containing 1% bovine serum albumin during the dissociation process, followed by centrifugation to remove the supernatant. Approximately 3×10^6^ spleen cells were collected in flow tubes for further experimentation.

*T lymphocyte sorting*

CD4^+^ T lymphocytes were isolated using the EasySep™ Mouse CD4^+^ T Cell Isolation Kit (STEMCELL, Canada). Spleens were processed into single-cell suspensions at a concentration of 1×10^8^ cells/mL. Rat serum was added to the sample followed by the Isolation Cocktail and incubated for 10 minutes. Streptavidin RapidSpheres™ were added, vortexed, and incubated for 2.5 minutes. Add the RoboSep™ buffer and place the flow tube into the magnetic incubator for 2.5 minutes, the CD4^+^ T cells were enriched.

Tregs were sorted using the EasySep™ Mouse CD4^+^CD25^+^ Regulatory T Cell Isolation Kit II (STEMCELL, Canada). CD4^+^ T cells enrichment with rat serum and CD4^+^ T-cell isolation mixture. Subsequently, Streptavidin RapidSpheres™ were added, and after magnet-based separation steps, and incubations, the enriched cell suspension was processed with FcR Blocker, CD25 Regulatory T Cell Positive Selection Cocktail, PE selection cocktail, and Dextran RapidSpheres™. Finally, the isolated Tregs were obtained following magnet-based separation and collection.

**1.7 q-PCR**

Total RNA was extracted from skeletal muscles using the Trizol method and Total RNA was extracted from cells using the kit (Magen, China). The cDNAs were synthesized from 1 μg of the total RNA in a 10 μl reaction system using a 5×All-in-one RT Mastermix (abm, China). Quantitation for individual target mRNA expression was performed with a CFX96™ Real-Time system (Bio-Rad, USA) using a ChamQ SYBR qPCR Master Mix (Vazyme, China). The amount of specific mRNA in each sample was calculated based on the cycle threshold (CT) values, which were standardized with the housekeeping gene GAPDH.

**1.8 Eukaryotic mRNA sequencing of skeletal muscles and Bioinformatics analysis**

Eukaryotic mRNA sequencing was performed by Shanghai Meiji Biomedical Technology Co. Total RNA was extracted using the Trizol method. The 2100 Bioanalyser (Agilent) and ND-2000 (NanoDrop Technologies) methods were used to test and ensure that the samples used were qualified (OD260/280=1.8~2.2, OD260/230≥2.0, RIN≥6.5, 28S:18S≥1.0, >1μg). RNA libraries were created using the TruSeqTM RNA Sample Preparation Kit (Illumina, San Diego, CA). The mRNA with poly-A tail was isolated and fragmented into small pieces of about 300 bp. Double-stranded cDNA was synthesized (Invitrogen, CA), repaired at the ends, and an A base was added at one end. The cDNA was PCR-amplified and sequenced on the Illumina HiSeq xten/NovaSeq 6000 platform with PE150 read length after screening and quantification.

Download IIM-related GSE datasets from the https://www.ncbi.nlm.nih.gov/geo/ database. Select the gene expression matrix file related to T cell exhaustion from the downloaded data and import it into R (version 4.2.2). Use the heatmap package to create a heatmap of T cell exhaustion-related genes. Import the prepared grouping file and gene matrix file into the GSEA software (version 4.3.2). Set the parameters with Gene sets database as h.all.v2022.1.Hs.symbols.gmt (hallmark) and Chips platform as Mouse_Gene_Symbol_Remapping_Human_Orthologs_MSigDB.v2022.1.Hs.chip. Run the program to obtain the results of the GSEA analysis.

**1.9 ELISA**

Inflammatory cytokine levels were measured in mouse serum and cell supernatants using ELISA assay kits (Ruixinbio or Meimian, China). The plate was set up with standard wells, zero wells, blank wells, and sample wells. Samples and controls were added to appropriate wells and treated with HRP-conjugated detection antibodies (37°C for 60 minutes). After washing, substrate(37°C for 15 minutes) and stop solutions (15 minutes) were added to the plate, followed by measuring the absorbance. A standard curve was created to calculate sample concentrations using a four-parameter logistic curve fitting based on OD values.

**1.10 Western blot**

Skeletal muscles were homogenized in a cold lysate containing protease inhibitors, phenylmethane sulfonyl fluoride, and phosphatase inhibitors. The denatured proteins were separated by 10% SDS-PAGE gel and then transferred to the PVDF membrane (Cytiva, China). The membranes were blocked (EpiZyme, China) and incubated overnight at 4 °C with the following antibodies: STAT3 (1:1000, CST), p-STAT3 (1:1000, CST), HIF-1α (1:2000, Proteintech Group), GAPDH (1:1000, Proteintech Group), and then incubated with HRP-secondary antibodies (1:4000, Proteintech Group) for 1 h at room temperature. Imaging was performed with an ECL chemiluminescent imaging system (Bio-Rad, USA), and the staining intensity of the bands was measured using ImageJ software.

**1.11** **Flow cytometry staining**

*Staining of Splenic T lymphocytes*

For Treg cell staining, 3×10^6^ splenic single cells were obtained following red blood cell lysis. The staining procedure involved the use of a viability dye (Zombie UV™, 1:100 for 15 min, Biolegend; or Dye eFluorTM780, 1:1000 for 1 min, Invitrogen), followed by extracellular antibodies (CD3, 1:50, Biolegend; CD4, 1:100, BD; CD25, 1:50, CD8, 1:50, PD1, 1:100, CTLA4, 1:100, TIM3, 1:100, LAG3, 1:50, Biolegend) for 30 min at 4℃. After fixation and permeabilization (20 min at 4℃, Invitrogen), the samples were incubated with FOXP3 antibody (1:100, 30 min at 4℃, Invitrogen).

For the Th1/2/17 cell staining, 3×10^6^ splenic single cells were taken and placed in 300μL of complete 1640 medium (containing 10% fetal bovine serum) with PMA (1:10000, Sigma), ionomycin (1:5000, Sigma), and Golgistop (1:1000, BD). The cells were then incubated for 4 hours in a 48-well plate. After washing, the cells were stained with viability dye (Dye eFluor™ 780, 1:1000, for 1 minute), followed by surface antibodies (CD3, 1:50, Biolegend; CD4, 1:100, BD; for 30 minutes at 4℃). Subsequently, the cells were fixed (for 20 minutes at 4℃, Invitrogen) and intracellular staining was performed using IFN-γ (1:100, BD), IL-4 (1:100, BD), IL-17A (1:50, BD), and p-STAT3 (1:50, Biolegend) antibodies for 30 minutes at 4℃.

*Staining of human peripheral blood T lymphocytes*

Layer freshly collected anticoagulated human peripheral blood above an equal volume of separation medium. Centrifuge at 700-800g with slow acceleration for 20 minutes. Carefully collect the middle buffy coat and transfer it to a new centrifuge tube. Following washing, take 3×10^6^ cells and conduct viability staining (Dye eFluor^TM^780, 1:1000, 15 min), then proceed with surface antibody staining (CD3, 1:50; CD4, 1:100; CD8, 1:50; PD1, 1:100; CTL4, 1:50; TIM3, 1:100; LAG3, 1:50, 30 min, 4°C, Biolegend) and fixation (30 min, 4°C).

After all staining procedures were completed, the samples were tested within 24 hours.

**1.12 Statistical analysis**

Data were presented as mean ± standard error (mean ± SEM) or median with interquartile range. SPSS 20 and GraphPad Prism 9 were used for statistical analyses and graphic production, respectively. Two-tailed t-test or Mann-Whitney test was used for statistical comparisons between two groups, and for comparisons between multiple groups, one-way ANOVA with Dunnett’s multiple comparison test or Brown–Forsythe–Welch ANOVA with Dunnett’s T3 multiple comparison test was used. Pearson correlation analysis or Spearman correlation analysis were used to assess the correlation between the two variables. Statistical significance was set at P<0.05.

[1] I.E. Lundberg, A. Tjärnlund, M. Bottai, et al., 2017 European League Against Rheumatism/American College of Rheumatology classification criteria for adult and juvenile idiopathic inflammatory myopathies and their major subgroups, Annals of the rheumatic diseases 76 (2017) 1955-1964.

[2] Y. Allenbach, A.L. Mammen, O. Benveniste, W. Stenzel, 224th ENMC International Workshop:: Clinico-sero-pathological classification of immune-mediated necrotizing myopathies Zandvoort, The Netherlands, 14-16 October 2016, Neuromuscular disorders : NMD 28 (2018) 87-99.

[3] J. Solomon, J.J. Swigris, K.K. Brown, Myositis-related interstitial lung disease and antisynthetase syndrome, Jornal brasileiro de pneumologia : publicacao oficial da Sociedade Brasileira de Pneumologia e Tisilogia 37 (2011) 100-109.

[4] L.G. Rider, V.P. Werth, A.M. Huber, et al., Measures of adult and juvenile dermatomyositis, polymyositis, and inclusion body myositis: Physician and Patient/Parent Global Activity, Manual Muscle Testing (MMT), Health Assessment Questionnaire (HAQ)/Childhood Health Assessment Questionnaire (C-HAQ), Childhood Myositis Assessment Scale (CMAS), Myositis Disease Activity Assessment Tool (MDAAT), Disease Activity Score (DAS), Short Form 36 (SF-36), Child Health Questionnaire (CHQ), physician global damage, Myositis Damage Index (MDI), Quantitative Muscle Testing (QMT), Myositis Functional Index-2 (FI-2), Myositis Activities Profile (MAP), Inclusion Body Myositis Functional Rating Scale (IBMFRS), Cutaneous Dermatomyositis Disease Area and Severity Index (CDASI), Cutaneous Assessment Tool (CAT), Dermatomyositis Skin Severity Index (DSSI), Skindex, and Dermatology Life Quality Index (DLQI), Arthritis care & research 63 Suppl 11 (2011) S118-157.
