# Supplementary material for: Adenosine A2B receptor activation regulates the balance between T helper 17 cells and regulatory T cells, and inhibits regulatory T cells exhaustion in experimental autoimmune myositis
Source: J Cachexia Sarcopenia Muscle. 2024 Sep 16;15(6):2460–75. doi: 10.1002/jcsm.13581 (PMC11634480; doi:10.1002/jcsm.13581)
Supplement: Supplementary file 3 — Data S3. Supplementary Figure S1–S10. [file JCSM-15-2460-s003.docx]

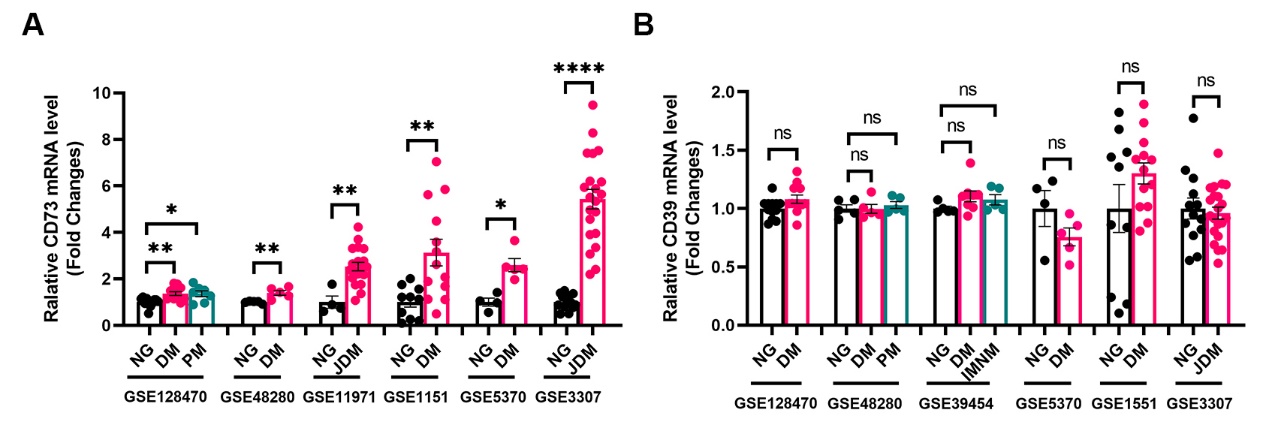


**Fig. S1 The mRNA levels of CD73 increased in affected skeletal muscle of patients with IIM**

A. The mRNA expression levels of CD73 in IIM subtypes of GSE128470, GSE48280, GSE11971, GSE1551, GSE5370, and GSE3307 datasets. vs. NG group. B. The mRNA expression levels of CD39 in IIM subtypes of GSE128470, GSE48280, GSE39454, GSE5370, GSE1551, and GSE3307 datasets. NG: normal group; DM: dermatomyositis; JDM: juvenile dermatomyositis; PM: polymyositis; IMNM: immune-mediated necrotizing myopathy; vs. NG group, *P<0.05, ** P<0.01, ****P<0.0001.


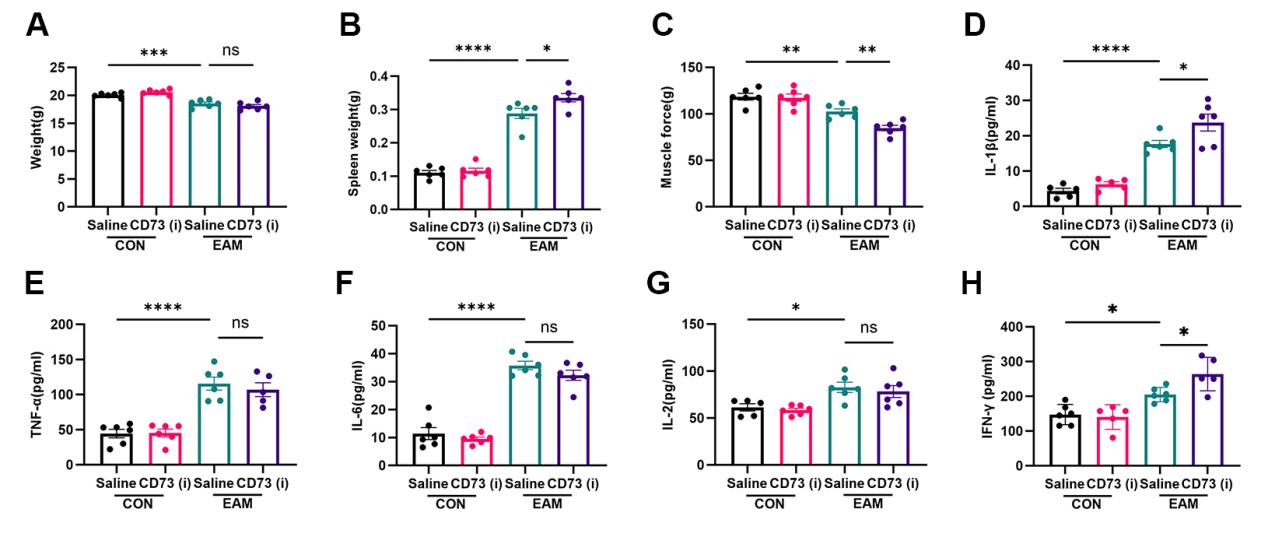


**Fig. S2 Effects of CD73 inhibition on body and spleen weight, muscle force, and inflammatory factors in mice**

Body weight (A), spleen weight (B), muscle force (C), and serum levels of IL-1β (D), TNF-α (E), IL-6 (F), IL-2 (G), and IFN-γ (H) levels in control and EAM mice after administration of the CD73 inhibitor or saline. CON: control. EAM: experimental autoimmune myositis; n=5-6; vs. EAM+Saline group, *P<0.05, **P<0.01, ***P<0.001, ****P<0.0001.


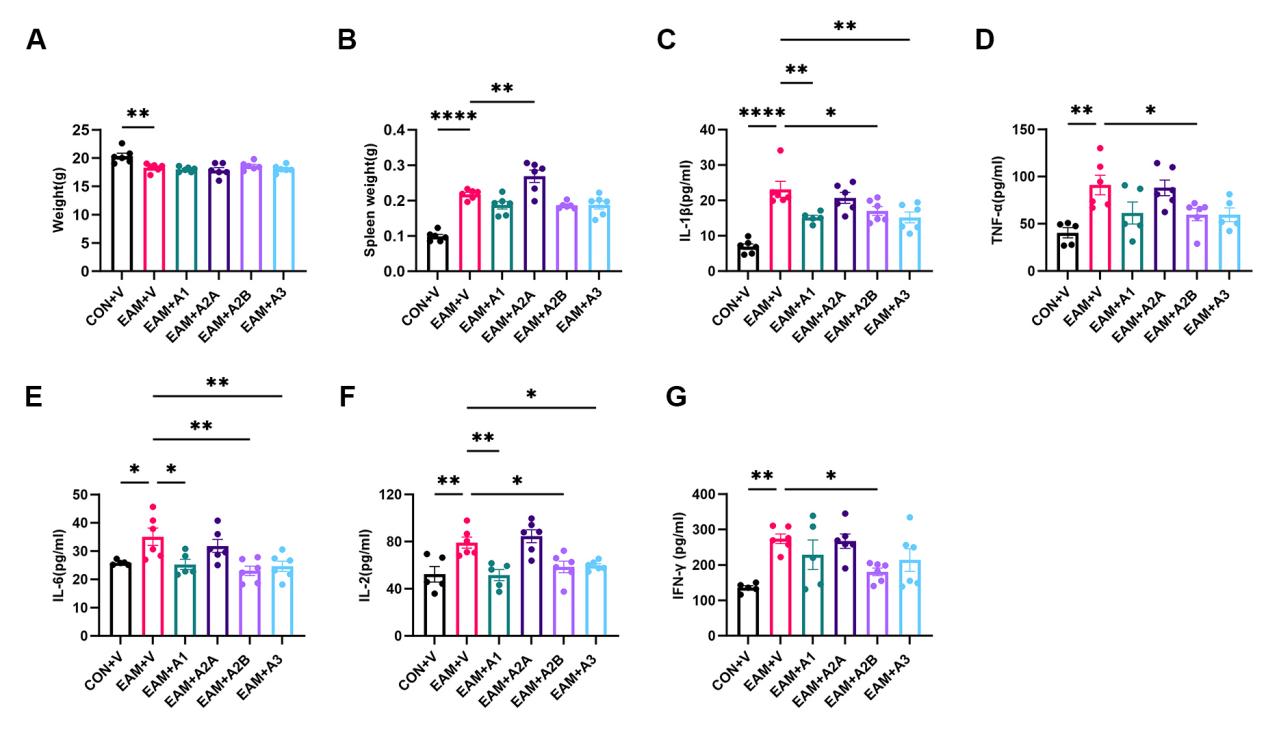


**Fig. S3 Effects of** **the adenosine receptor agonists on body and spleen weight, and inflammatory factors in mice**

Body weight (A), spleen weight (B), and serum levels of IL-1β (C), TNF-α (D), IL-6 (E), IL-2 (F), and IFN-γ (G) in control and EAM mice after administration of the adenosine receptor agonists or vehicle. CON: control. EAM: experimental autoimmune myositis; V: vehicle; n=5-6; vs. EAM+V group, *P<0.05, **P<0.01, ****P<0.0001.


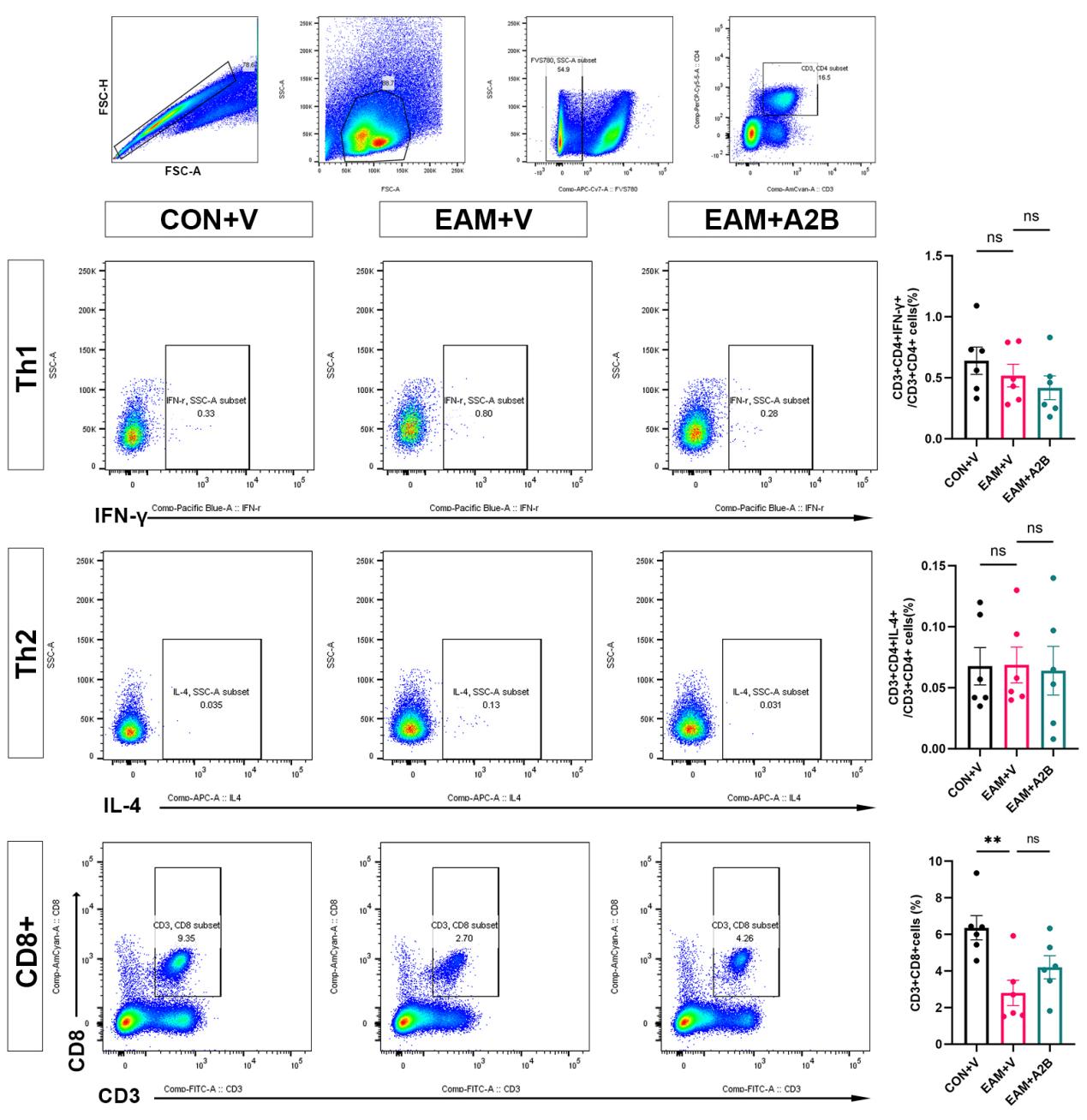


**Fig. S4** **Activation of adenosine A2B receptor does not affect splenic Th1, Th2, and** **CD8^+^T cells in EAM mice.**

Flow staining scheme, scatter plots, and proportion of splenic Th1, Th2, and CD8^+^T cells in control and EAM mice after administration of the adenosine A2B receptor agonist or vehicle. CON: control. EAM: experimental autoimmune myositis; V: vehicle; n=6; vs. EAM+V group. **P<0.01.


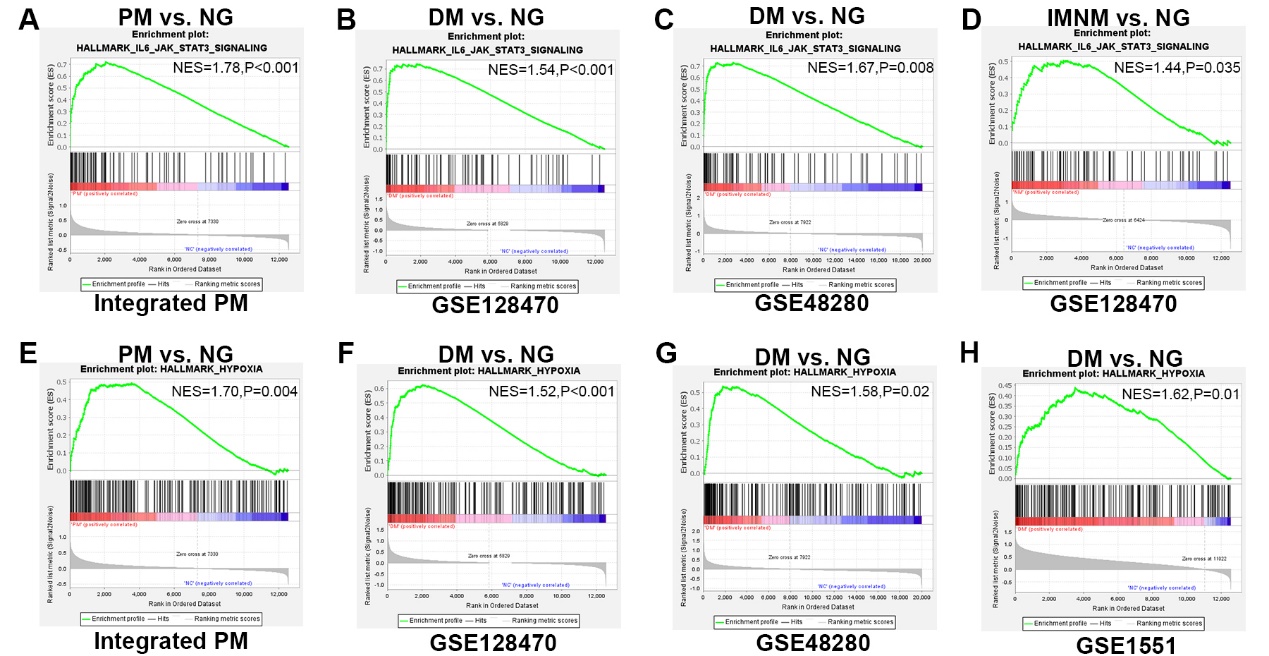


Fig. S5 GSEA of IL-6/STAT3 and HYPOXIA signaling pathways in affected skeletal muscle in patients with IIM

GSEA of IL-6/STAT3 signaling pathway in patients with PM (A), DM (B-C), and IMNM (D). GSEA of HYPOXIA pathway in patients with PM (E) and DM (F-H). PM: polymyositis; DM: dermatomyositis; INMN: immune-mediated necrotizing myopathy; GSEA: Gene Set Enrichment Analysis; NES: Normalized Enrichment Score.


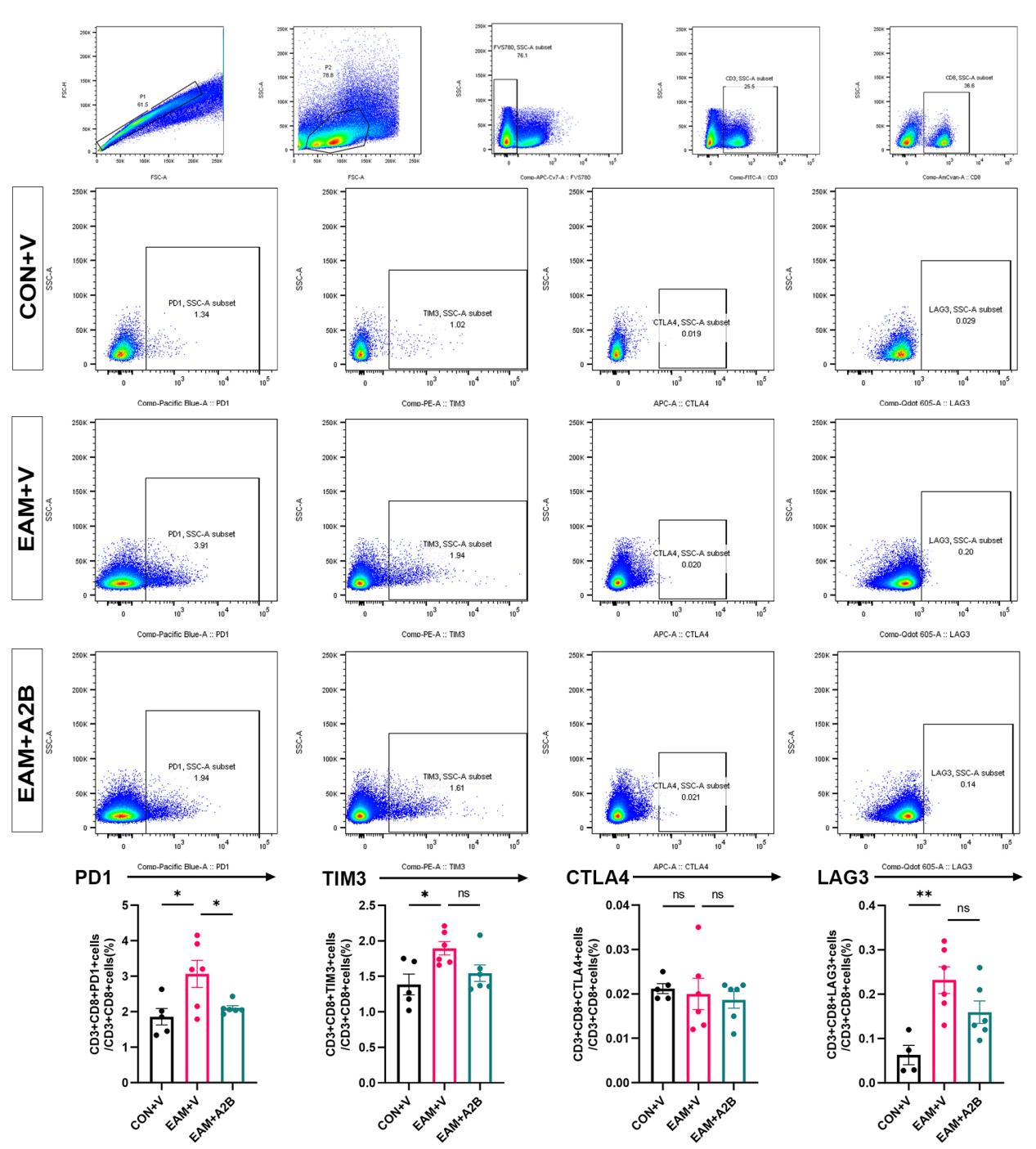


**Fig. S6** **Effect of adenosine A2B receptors activation on splenic CD8^+^ T cell** **exhaustion in EAM mice**

Flow scheme, scatter plots, and proportions of exhausted CD8^+^ T cells (splenic PD1^+^CD8^+^ T cells, TIM3^+^CD8^+^ T cells, CTLA4^+^CD8^+^ T cells, and LAG3^+^CD8^+^ T cells) in control and EAM mice after administration of the adenosine A2B receptor agonist or vehicle. PD1: Programmed cell death protein-1; TIM3; T-cell immunoglobulin and mucin-domain containing-3; CTLA4; Cytotoxic T-lymphocyte-associated protein-4; LA3; Lymphocyte-activation gene-3; CON: control; EAM: experimental autoimmune myositis; n=4-6; V: Vehicle; vs. EAM+V group, *P<0.05, **P<0.01.


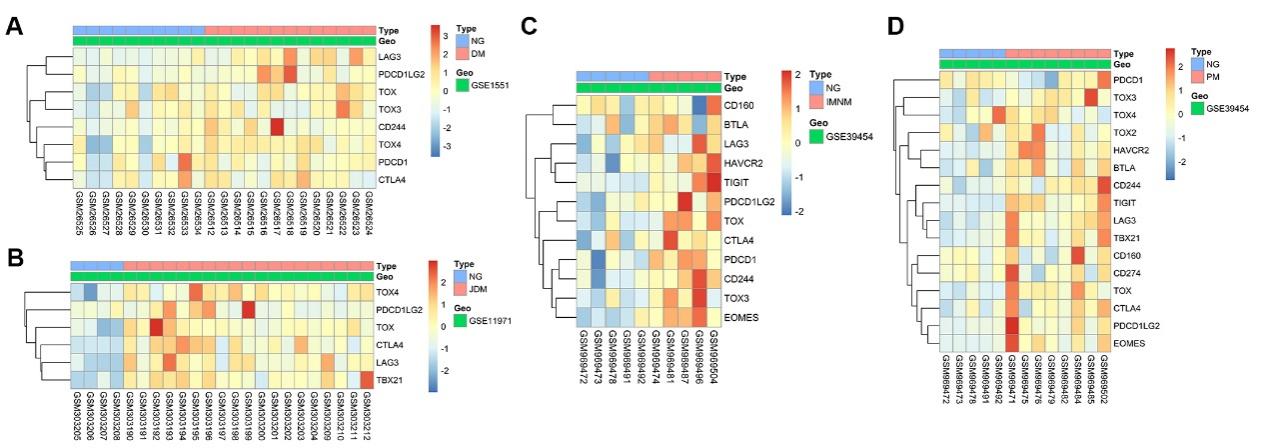


Fig. S7 Elevated mRNA levels of exhaustion-related genes in affected skeletal muscles in IIM datasets.

Heatmaps of exhaustion-related gene expression in IIM subtypes in GSE1551 (A), GSE11971 (B), and GSE39454 (C, D) datasets. PM: polymyositis; DM: dermatomyositis; JDM: Juvenile dermatomyositis; INMN: immune-mediated necrotizing myopathy, NG: normal group.


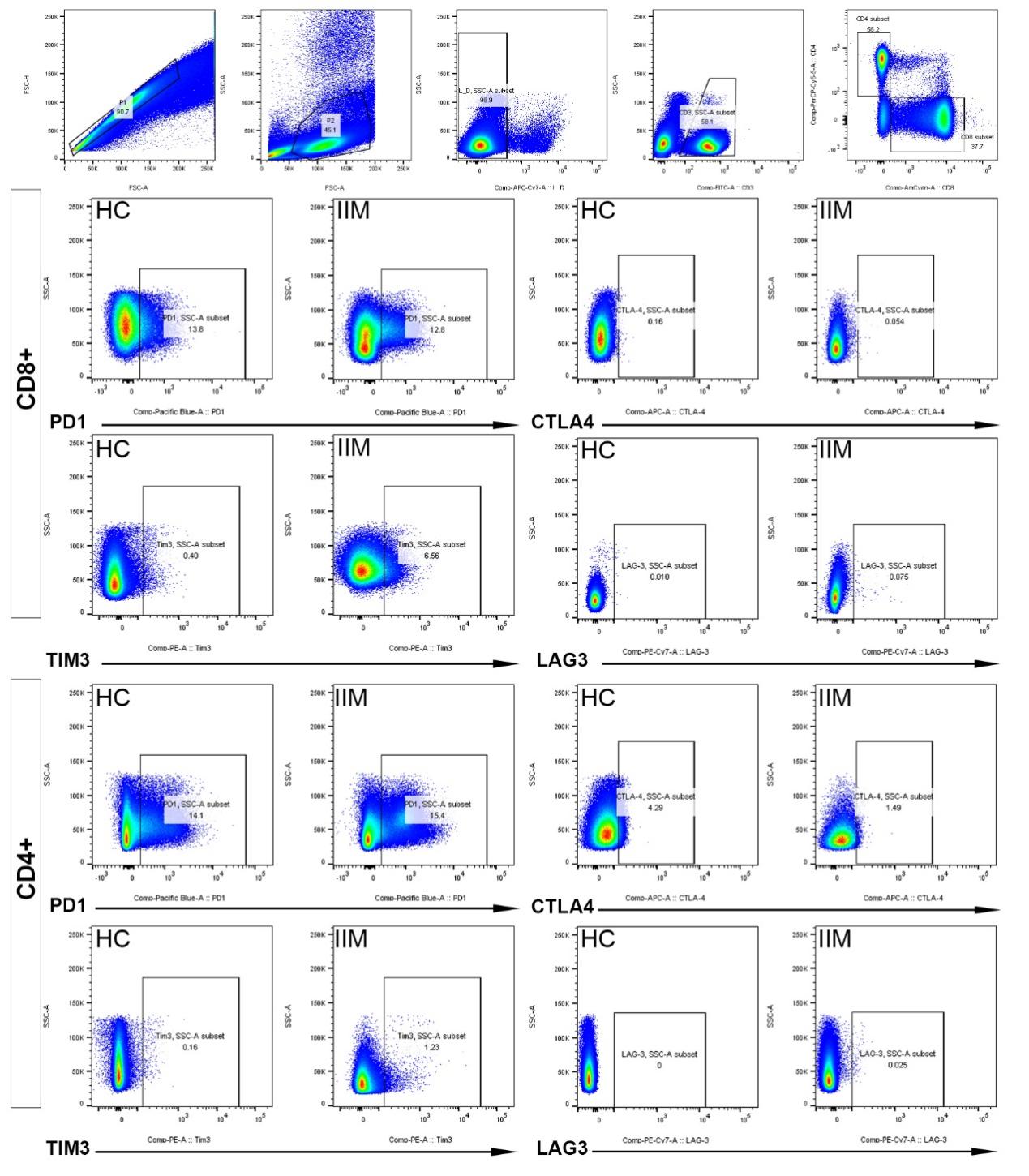


**Fig. S8 Flow scatter plots of exhausted CD8^+^ and CD4^+^ T cells in peripheral blood of patients with IIM**

Flow scheme, scatter plots of exhausted CD8^+^ T cells (PD1^+^CD8^+^ T cells, TIM3^+^CD8^+^ T cells, CTLA4^+^CD8^+^ T cells, and LAG3^+^CD8^+^ T cells) and exhausted CD4^+^ T cell (PD1^+^CD4^+^ T cells, TIM3^+^CD4^+^ T cells, CTLA4^+^CD4^+^ T cells, and LAG3^+^CD4^+^ T cells) in peripheral blood of HC (n=30) and patients with IIM (n=63). PD1: Programmed cell death protein-1; TIM3: T-cell immunoglobulin and mucin-domain containing-3; CTLA4: Cytotoxic T-lymphocyte-associated protein-4; LAG3: Lymphocyte-activation gene-3; HC: healthy control; IIM: idiopathic inflammatory myopathy.


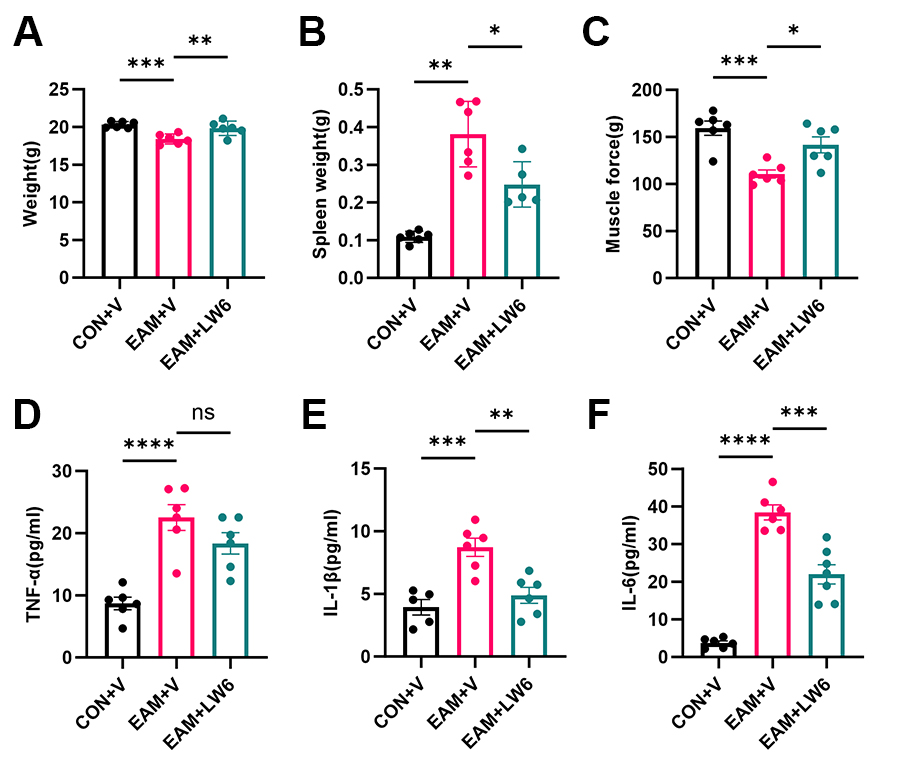


Fig. S9 Effects of HIF-1α inhibition on body and spleen weight, muscle force, and inflammatory factors in mice

Body weight (A), spleen weight (B), muscle force (C) and serum TNF-α (D), IL-1β (E) and IL-6 (F) levels in control and EAM mice after administration of the HIF-1α inhibitor or vehicle. CON: control; EAM: experimental autoimmune myositis; V: vehicle; LW6: a HIF-1α inhibitor; n=6; vs. EAM+V group, *P<0.05, **P<0.01, ***P<0.001, ****P<0.0001.

**
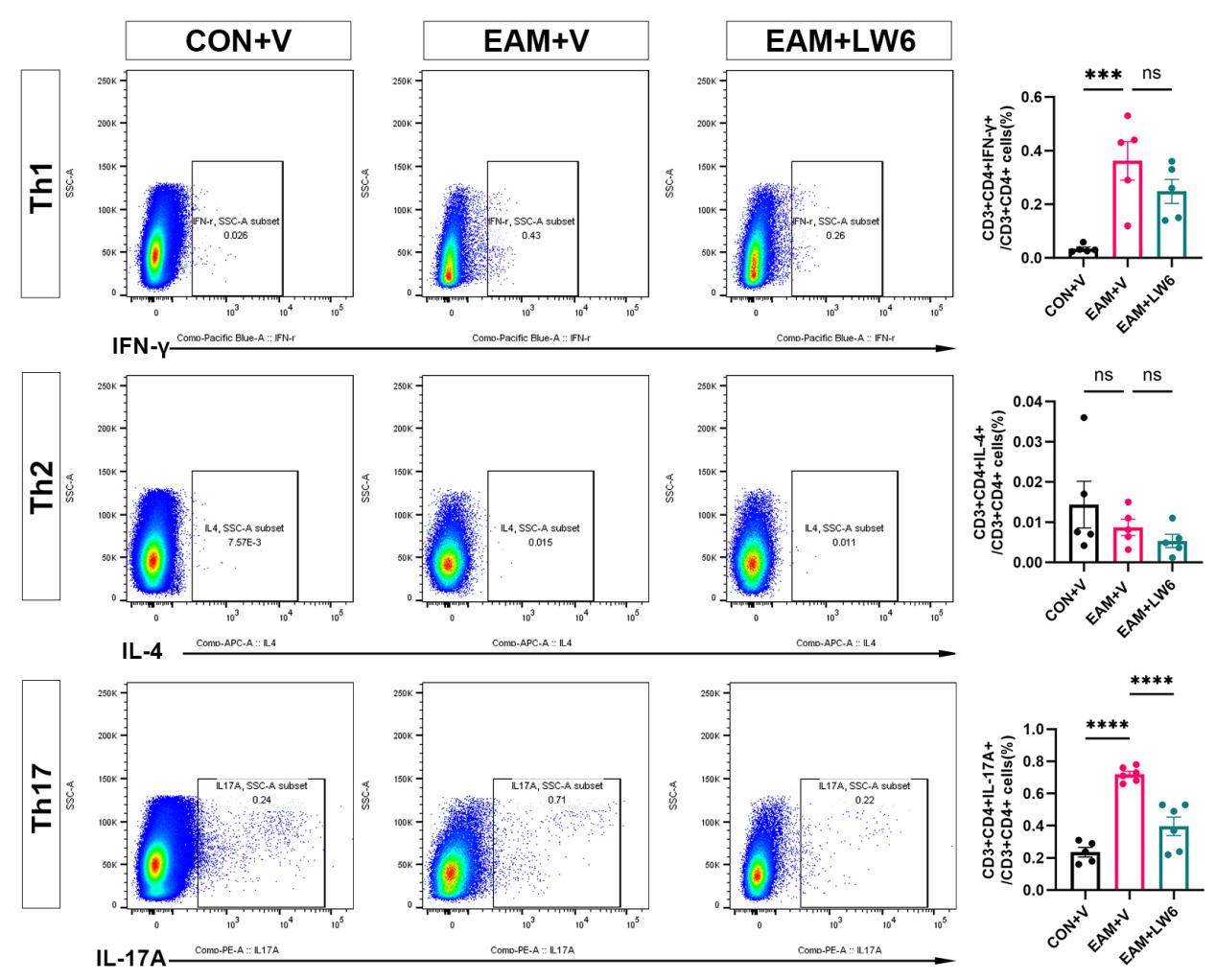
Fig. S10 Effects of HIF-1α inhibition on splenic Th1, Th2, and Th17 in EAM mice**

Flow scatter plots and proportions of splenic Th1, Th2 and Th17 in mice. CON: control; EAM: experimental autoimmune myositis; V: vehicle; LW6: a HIF-1α inhibitor; n=5-6；vs. EAM+V group, ***P<0.001, ****P<0.0001.
